# Supplementary material for: Candidate pathogenicity islands in the genome of ‘Candidatus Rickettsiella isopodorum’, an intracellular bacterium infecting terrestrial isopod crustaceans
Source: PeerJ. 2016 Dec 21;4:e2806. doi: 10.7717/peerj.2806 (PMC5181103; doi:10.7717/peerj.2806)
Supplement: Supplemental Information 1 [file peerj-04-2806-s001.docx]

**Supplementary Figure 1.** Regions predicted by REAPR to contain assembly errors align to the *Rickettsiella grylli* NZ_AAQJ00000000 in one continuous block, suggesting that these regions are indeed assembled correctly in spite of REAPR's error predictions. The example below shows a 4000-bp region from our *R. grylli* assembly predicted to contain an assembly error by REAPR (on contig 828, bases 2337 to 2585) aligns perfectly to the reference *R. grylli* genome. Bases 1 to 4000 all aligned perfectly, but only a ~1500 bp region flanking the predicted assembly error is shown below for brevity.

Query= contig_828:1-4000

Length=4000

Score E

Sequences producing significant alignments: (Bits) Value

contig_2 7199 0.0

> contig_2

Length=1566411

Score = 7199 bits (3898), Expect = 0.0

Identities = 3967/4001 (99%), Gaps = 2/4001 (0%)

Strand=Plus/Plus

Query 1500 AATTGAGAAAAACGACCCCGTAGTGGTTTTGGAAATGCAGATTCTAAAACGCGTTTTAAA 1559

|||||||||||||| |||||||||||||||||||||||||||||||||||||||||||||

Sbjct 1148400 AATTGAGAAAAACGGCCCCGTAGTGGTTTTGGAAATGCAGATTCTAAAACGCGTTTTAAA 1148459

Query 1560 TAATCTTCCTCTAAGGAATGTTCTTCGAGTAATTCTGTTTTTACCCACATTTTGGTATAA 1619

||||||||||||||||||||||||||||||||||||||||||||||||||||||||||||

Sbjct 1148460 TAATCTTCCTCTAAGGAATGTTCTTCGAGTAATTCTGTTTTTACCCACATTTTGGTATAA 1148519

Query 1620 GCAAGTAATACAGCAATTTCAGGGCTGGTAAGCCCTTTACCCATTGCCTTTCTTTCTAAT 1679

||||||||||||||||||||||||||||||||||||||||||||||||||||| ||||||

Sbjct 1148520 GCAAGTAATACAGCAATTTCAGGGCTGGTAAGCCCTTTACCCATTGCCTTTCTCTCTAAT 1148579

Query 1680 AAGGCTTTTTCATCCGGAAGAAACTCTAAAGCACGATTGAGTTTACCATGCTGCTCAAGT 1739

||||||||||||||||||||||||||||||||||||||||||||||||||||||||||||

Sbjct 1148580 AAGGCTTTTTCATCCGGAAGAAACTCTAAAGCACGATTGAGTTTACCATGCTGCTCAAGT 1148639

Query 1740 TCTTGAATGTAACGACGATGAAATTCCAATTCTTGCTGAGCATGCATGGCCGCTAAACTA 1799

||||||||||||||||||||||||||||||||||||||||||||||||||||||||||||

Sbjct 1148640 TCTTGAATGTAACGACGATGAAATTCCAATTCTTGCTGAGCATGCATGGCCGCTAAACTA 1148699

Query 1800 ATCGTACGCGTTTGACAATAATTGTCATAAAGAACCAGTTTAGCAATCTCATCGGTCATT 1859

||||||||||||||||||||||||||||||||||||||||||||||||||||||||||||

Sbjct 1148700 ATCGTACGCGTTTGACAATAATTGTCATAAAGAACCAGTTTAGCAATCTCATCGGTCATT 1148759

Query 1860 TCCGCTAATAACGTATTTCGTTCTTCGAAACTCATTTCACCGGCAGTAACAACGGCATTT 1919

||||||||||||||||||||||||||||||||||||||||||||||||||||||||||||

Sbjct 1148760 TCCGCTAATAACGTATTTCGTTCTTCGAAACTCATTTCACCGGCAGTAACAACGGCATTT 1148819

Query 1920 AATAAAATTTTACAATTGACTTCATGATCAGAACAATCAACACCTGCAGAATTATCAATA 1979

||||||||||||||||||||||||||||||||||||||||||||||||||||||||||||

Sbjct 1148820 AATAAAATTTTACAATTGACTTCATGATCAGAACAATCAACACCTGCAGAATTATCAATA 1148879

Query 1980 AAGTCGGTATAAATTAATCCGCCATTTAAAGCATATTCGACTCGACCCAACTGCGTTAGA 2039

||||||||||||||||||||||||||||||||||||||||||||||||||||||||||||

Sbjct 1148880 AAGTCGGTATAAATTAATCCGCCATTTAAAGCATATTCGACTCGACCCAACTGCGTTAGA 1148939

Query 2040 CCAAGATTACCCCCCTCAGCGACAATACGACAGCGCAATTCTTTCGCGTCAATCCGTAAA 2099

|||||||||||||||||||||||||||||||| |||||||||||||||||||||||||||

Sbjct 1148940 CCAAGATTACCCCCCTCAGCGACAATACGACAACGCAATTCTTTCGCGTCAATCCGTAAA 1148999

Query 2100 TTATCATTGGTTCTATCGCCTACGTCGGCATTTCGTTCATTGGACGCTTTCACATACGTG 2159

||||||||||||||||||||||||||||||||||||||||||||||||||||||||||||

Sbjct 1149000 TTATCATTGGTTCTATCGCCTACGTCGGCATTTCGTTCATTGGACGCTTTCACATACGTG 1149059

Query 2160 CCAATCCCTCCGTTCCACAGTAAATCGACATTTGCTTTTAATAGTGCTCGAATTAATCCA 2219

||||||||||||||||||||||||||||||||||||||||||||||||||||||||||||

Sbjct 1149060 CCAATCCCTCCGTTCCACAGTAAATCGACATTTGCTTTTAATAGTGCTCGAATTAATCCA 1149119

Query 2220 TCGGGCGCTATAGAATCTTGATGAATATCTAATAATTTTTTAATTTCACTCGATAAGACA 2279

||||||||||||||||||||||||||||||||||||||||||||||||||||||||||||

Sbjct 1149120 TCGGGCGCTATAGAATCTTGATGAATATCTAATAATTTTTTAATTTCACTCGATAAGACA 1149179

Query 2280 ATCGATTTTTGTGAGCGTAGGAAAATTCCCCCTCCTTTAGAAAGCAAATGAGCATTGTAA 2339

||||||||||||||||||||||||||||||||||||||||||||||||||||||||||||

Sbjct 1149180 ATCGATTTTTGTGAGCGTAGGAAAATTCCCCCTCCTTTAGAAAGCAAATGAGCATTGTAA 1149239

Query 2340 TCTTTCCATGTAGAACGAGGTAAATGAAATAAACGCTTTCGTTCTTCGAAACTTTTTTCT 2399

||||||||||||||||||||||||||||||||||||||||||||||||||||||||||||

Sbjct 1149240 TCTTTCCATGTAGAACGAGGTAAATGAAATAAACGCTTTCGTTCTTCGAAACTTTTTTCT 1149299

Query 2400 GGACAGGGATTGGGATCAATAAAGATGTGCAGGTGATTAAATGCGGCAACAAGCTTAATA 2459

||||||||||||||||||||||||||||||||||||||||||||||||||||||||||||

Sbjct 1149300 GGACAGGGATTGGGATCAATAAAGATGTGCAGGTGATTAAATGCGGCAACAAGCTTAATA 1149359

Query 2460 TGACGCGATAGTAACATTCCATTACCAAAAACATCGCCTGACATATCACCAATACCCACC 2519

||||||||||||||||||||||||||||||||||||||||||||||||||||||||||||

Sbjct 1149360 TGACGCGATAGTAACATTCCATTACCAAAAACATCGCCTGACATATCACCAATACCCACC 1149419

Query 2520 ACAGTAAAATCATCTTTATCCGGATTTAATCCAAGGGCTCTACAATGTCTTCTAACCGAT 2579

|| |||||||||||||||||||||||||||||||||||||||||||||||||||||||||

Sbjct 1149420 ACCGTAAAATCATCTTTATCCGGATTTAATCCAAGGGCTCTACAATGTCTTCTAACCGAT 1149479

Query 2580 TCCCATGCCCCTCGAGCCGTAATCCCCAtttttttATGATCGTAGCCCACGCTTCCACCC 2639

||||||||||| ||||||||||| |||||||||||||||||||| |||||||||||||||

Sbjct 1149480 TCCCATGCCCCCCGAGCCGTAATTCCCATTTTTTTATGATCGTAACCCACGCTTCCACCC 1149539

Query 2640 GAAGCAAAGGCATCACCCAACCAAAAATTGTATTCCGCTGCGATAGCATTGGCGATATCT 2699

||||||||||| ||||||||||||||||||||||||||||||||||||||||||||||||

Sbjct 1149540 GAAGCAAAGGCGTCACCCAACCAAAAATTGTATTCCGCTGCGATAGCATTGGCGATATCT 1149599

Query 2700 GAGAAACTTGCTGTACCTTTGTCTGCCGCAACCACTAAATAAGGATCGTCCTCGTCGTAA 2759

||||||||||||||||||||||||||||||||||||||||||||||||||||||||||||

Sbjct 1149600 GAGAAACTTGCTGTACCTTTGTCTGCCGCAACCACTAAATAAGGATCGTCCTCGTCGTAA 1149659

Query 2760 CGTACTACGTTTTCAGGATGTATAATGCTATTATTTTTCAAATTATCCGTTAAATCTAAC 2819

||||||||||||||||||||||||||||||||||||||||||||||||||||||||||||

Sbjct 1149660 CGTACTACGTTTTCAGGATGTATAATGCTATTATTTTTCAAATTATCCGTTAAATCTAAC 1149719

Query 2820 AAGCCTCGCATAAACGTTTGATAACACGAAATCACTTCCTTCATCACTGCTTCGCGATCA 2879

||||||||||||||||||||||||||||||||||||||||||||||||||||||||||||

Sbjct 1149720 AAGCCTCGCATAAACGTTTGATAACACGAAATCACTTCCTTCATCACTGCTTCGCGATCA 1149779

Query 2880 GCATTTTCATAGAGCTGCTTACAAACAAAACCACCTTTAGCACCGGCAGGCACTATCACC 2939

||||||||||||||||||||||||||||||||||||||||||||||||||||||||||||

Sbjct 1149780 GCATTTTCATAGAGCTGCTTACAAACAAAACCACCTTTAGCACCGGCAGGCACTATCACC 1149839

Query 2940 GCATTCTTGACCTGCTGTGCCTTCATCAAACCTAATATTTCTGTCCGAAAATCTTCTCTT 2999

||||||||||||||||||||||||||||||||||||||||||||||||||||||||||||

Sbjct 1149840 GCATTCTTGACCTGCTGTGCCTTCATCAAACCTAATATTTCTGTCCGAAAATCTTCTCTT 1149899

Query 3000 CTGTCAGACCAACGAATACCACCACGGGCTACCTTCGCAGCTCTTAGATGAACGGCTTCA 3059

||||||||||||||||||||||||||||||||||||||||||||||||||||||||||||

Sbjct 1149900 CTGTCAGACCAACGAATACCACCACGGGCTACCTTCGCAGCTCTTAGATGAACGGCTTCA 1149959

**Supplementary Figure 2.** Read alignments do not support assembly errors predicted by REAPR in the *Rickettsiella grylli* assembly obtained from *Trachelipus rathkei*. Predicted assembly errors do have lower coverage than other regions, but raw sequence reads still align to and span these regions, and there is no evidence of discordantly mapped reads. The example plots below were generated by IGV.

A. Contig 828
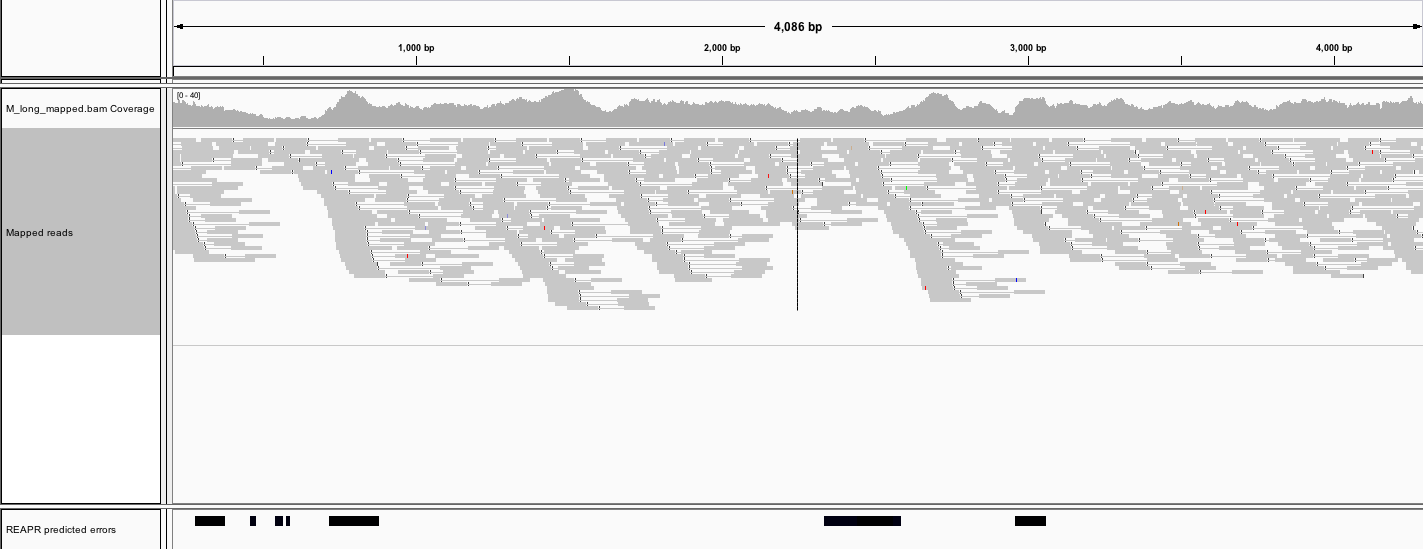


B. Contig 844


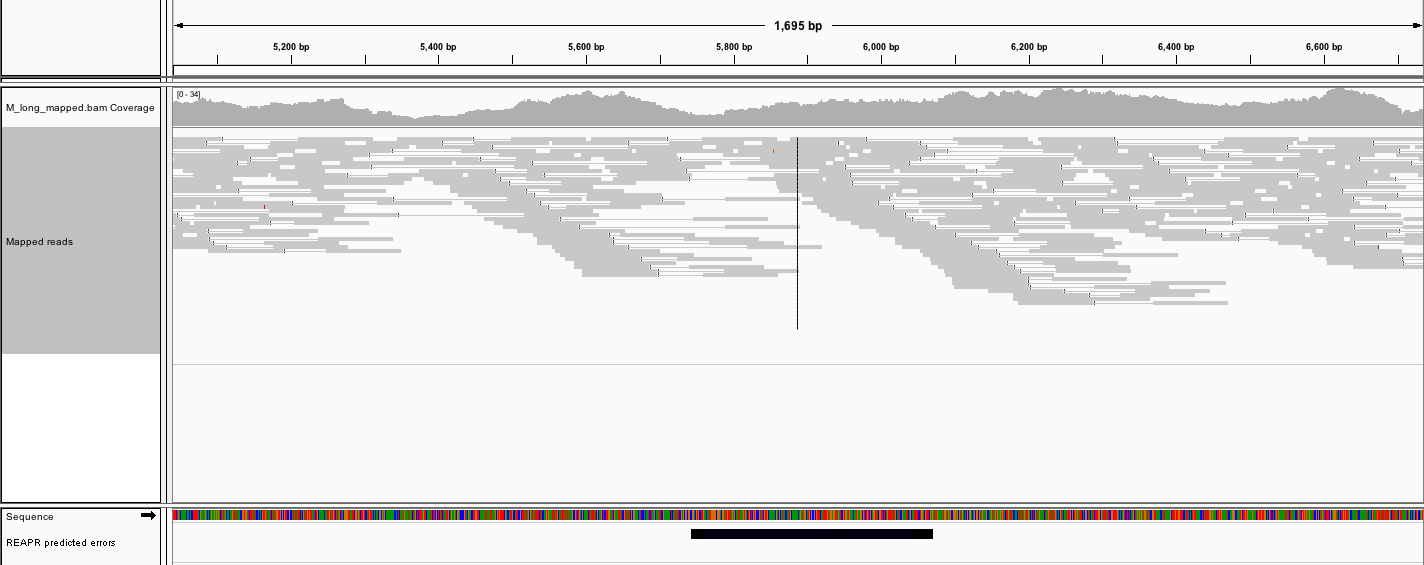


C. Contig 847


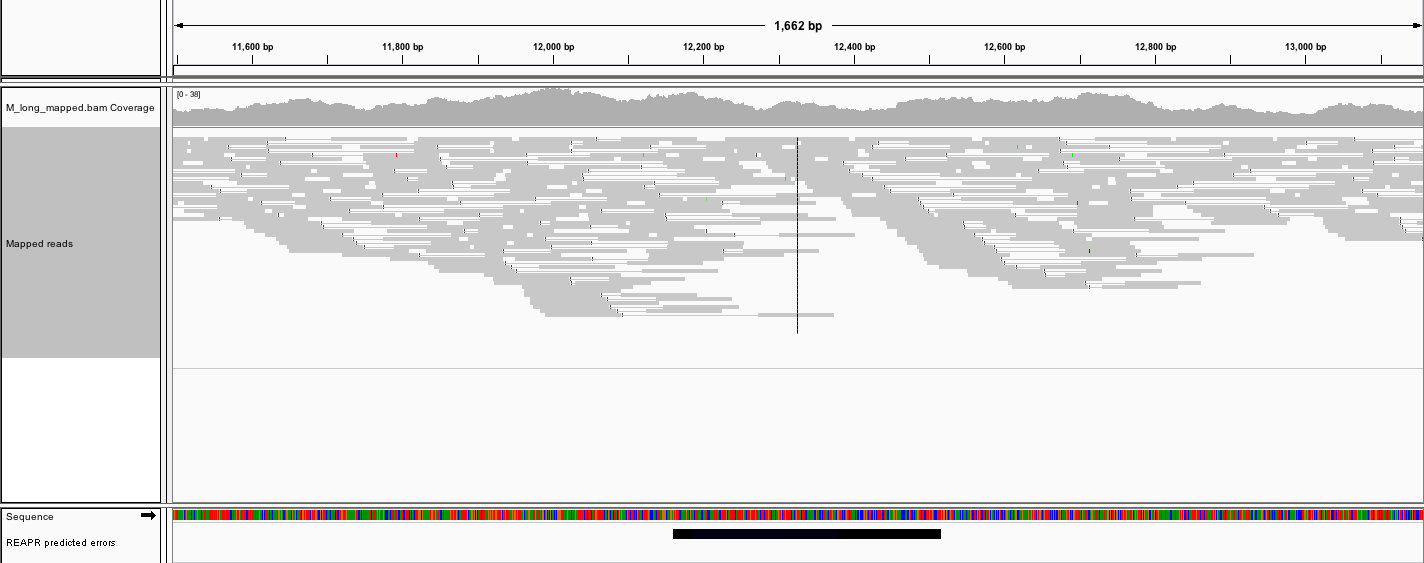


**Supplementary Figure 3.** Regions predicted by REAPR to contain assembly errors align to the *Rickettsiella grylli* NZ_AAQJ00000000 in one continuous block, suggesting that these regions are indeed assembled correctly in spite of REAPR's error predictions. The example below shows a 3000-bp region from our *R. isopodorum* assembly predicted to contain an assembly error by REAPR (on contig 196, bases 65377 to 65611) aligned to the reference *R. grylli* genome. Bases 64000 to 67000 all aligned perfectly, albeit with a fair number of substitutions and a few small indels because of the higher divergence between *R. grylli* and *R. isopodorum*.

Query= contig_196:64000-67000

Length=3001

Score E

Sequences producing significant alignments: (Bits) Value

contig_2 1981 0.0

> contig_2

Length=1566411

Score = 1981 bits (2196), Expect = 0.0

Identities = 2218/2956 (75%), Gaps = 35/2956 (1%)

Strand=Plus/Plus

Query 33 TTTTATGCATTTAAGTCAACTATTAATAGGGATTGGTCTTTCAGTAACGGCAGATCCTAT 92

|||||||||||| || ||| ||||| || || | ||||| || | | ||||||||

Sbjct 537276 TTTTATGCATTTGAGCCAATTATTACGCGGAATGAATATTTCAATAGCAACTGATCCTAT 537335

Query 93 TATTAAAGGGCTTTGCCAAGATAGTCGTCAAGTAAAGCCAGGCGATCTATTCTTTGCCTA 152

||||||||| || || ||||||||||| || ||||||| || |||||||||||||| ||

Sbjct 537336 TATTAAAGGACTCTGTCAAGATAGTCGGCAGATAAAGCCCGGTGATCTATTCTTTGCTTA 537395

Query 153 TCCTGGATTGGATCGTGATGGCCGTCACTTCATACCCGAGGCGATTGCTAAAGGTGCGGC 212

| || || | ||||||| || | || || || || ||| |||||||| ||

Sbjct 537396 TAAAGGTTTAAAAAGTGATGGACGCTATTTTATTAAGGATGCCATTAAAAAAGGTGCAGC 537455

Query 213 GGCTATTCTATTTGAATCGGAGGGGAATTCTGTGGAGCGG---AGTTCGTCATTCATTCC 269

|||||| || | ||| | || | |||| | | | | || || | |||||

Sbjct 537456 AGCTATTTTAGTGGAACCCGACGAAAATTATACGATACATTTAAAATCATCCTCTATTCC 537515

Query 270 TATATTACCGCTTAATCATTTAACCGCACAATTAGGACCTATAGCCGCCCGATTTTATGA 329

|||| || || | | |||||||| |||| |||||| | | || || ||||||||||

Sbjct 537516 TATAATAGCGTTGCACCATTTAACATCACAGTTAGGATCACTTGCTGCACGATTTTATGG 537575

Query 330 TTATCCGAGTCGTTATTTGCCGGTAATAGGCATTACGGGTACGAATGGTAAAACGTCCTG 389

|||||||||||| ||| | || || || || || || ||||||||||||||||| || ||

Sbjct 537576 TTATCCGAGTCGCTATCTACCCGTTATCGGAATAACTGGTACGAATGGTAAAACATCGTG 537635

Query 390 TACTCACTTTTTAGCGGATAGTTTGCAGCAGTTACAAAAACCTTGTGGTGTCATCGGTAC 449

||| || ||||| || ||||| ||||| |||||||| | | |||||||| ||||| ||

Sbjct 537636 TACCCATTTTTTGGCAGATAGCTTGCAAAAGTTACAACGATCGTGTGGTGTTATCGGCAC 537695

Query 450 CTTAGGAAATGGTTTTTATGGTGATTTAAAGCCGGGTCAATTAACCACACCGGATGCCAT 509

|||||| || |||||||||||| ||||||| | | ||||||||| ||||||||||||||

Sbjct 537696 CTTAGGCAACGGTTTTTATGGTCATTTAAAACGCGCTCAATTAACGACACCGGATGCCAT 537755

Query 510 TGAGTTACAGCAATTGCTGGCAAATTTTCGGGATAAGCAAGCACAAGCCGTAGTGATGGA 569

|| |||| ||||| || ||| |||||| | | | || |||||| | || || |||||

Sbjct 537756 CGAACTACAACAATTACTCGCAGCTTTTCGCGCTCAACACGCACAAACGGTTGTTATGGA 537815

Query 570 AGTGTCTTCACATCGATTAGCCCAACAGCGTTTAAATGGAACAGAATTTTCTGTTGCCGC 629

||| || || || |||| || ||| |||||||||||||||| |||||||| || |||||

Sbjct 537816 AGTTTCCTCGCACAGATTGGCTCAAAAGCGTTTAAATGGAACTGAATTTTCGGTCGCCGC 537875

Query 630 ATTTACCAATTTGACACGTGATCATCTTGATTATCATGAATCGATGAGTGCTTATGCCCA 689

||| || |||||||| |||||||| | || |||||| || | ||| ||||||||| |

Sbjct 537876 ATTGACTAATTTGACGCGTGATCACTTAGACTATCATAAAACAATGGATGCTTATGCAAA 537935

Query 690 AGCTAAACGTTCTATTTTTGATTTGCCGGGTGTGCAGCAGGCAATATTAAATGCCGATGA 749

|| || || ||| ||||||| || || ||||| || || | ||||||||||||||

Sbjct 537936 GGCCAAGCGCTCTTTTTTTGAATTACCCGGTGTCCAACACATTGTTTTAAATGCCGATGA 537995

Query 750 TCCTTACGCACAATTATGGTTAACTGAGCTTGCTGAACAATTACCGGTGTATACTTATTC 809

|||||||||||||||||||||||||||| ||| ||||| | || | | |||||||||

Sbjct 537996 TCCTTACGCACAATTATGGTTAACTGAGTTTGAGAAACAACTCCCTATCTCTACTTATTC 538055

Query 810 TTTACATAAGCCACAAGCGGCATGGCCACATATTCCACAGATCACAGTaaaaaaaTTTGA 869

||| | || |||||| ||| |||||||||||||| |||| ||||||||||||||

Sbjct 538056 GTTAAAACGTCCTCAAGCGCGTTGGTCACATATTCCACAGGTCACGGTAAAAAAATTTGA 538115

Query 870 TTTTAATCAGCAAGGTTTACGGGCAGAAATTGATACGCCCTGGGGCGAAGTATTTATCGA 929

||||||||| || || ||| |||||||||||||||||||||||| || |||||||| ||

Sbjct 538116 TTTTAATCAACAGGGACTACAGGCAGAAATTGATACGCCCTGGGGAGACGTATTTATTGA 538175

Query 930 AAATCGATTTTTAATGGGACCGTTTAATTTAAGTAATTTGTTATTGGTTTTGACGATATT 989

||||| ||||||||||| |||||||||||||||||||||| || || || || | |

Sbjct 538176 AAATCAGTTTTTAATGGGCGCGTTTAATTTAAGTAATTTGTTGTTAGTATTAACAGTGCT 538235

Query 990 GAAAAATTTACATTTTTCTTTAGCAGAAATTTCGCAAGTGATCTCGAAGTTAAAAGGTGT 1049

||||| |||||||||||||| || |||||||||||||| | ||| |||||| | |

Sbjct 538236 GAAAAGCTTACATTTTTCTTTGGCCGAAATTTCGCAAGTTTTATCGCGATTAAAAAGCGC 538295

Query 1050 AAAAGGGCGTATGCAAGCTTTTCATGTACCTGGAAAAGCCTTAGTTGTCGTAGATTATGC 1109

||||| || |||||| |||||| || |||||||| | ||||| || ||||| ||

Sbjct 538296 TAAAGGACGCATGCAAACTTTTCGAGTGAAAGGAAAAGCAATCGTTGTTGTGGATTACGC 538355

Query 1110 GCACACGCCGGATGCTTTGCAGCAAGTGTTGCGTGCTTTACGACCGCATTGTGCTGGTGA 1169

||| || || ||||| | ||||| || |||| || ||| | |||||||| || |

Sbjct 538356 GCATACACCCGATGCGCTACAGCAGGTTTTGCAGGCGTTAAGGTCGCATTGTCGAGGGCA 538415

Query 1170 GTTATATTGTTTATTTGGTTGTGGTGGTGATCGAGATAAAGGTAAACGGCCCTTAATGGC 1229

| |||||||||||||||||||| || ||||| |||||||| ||||| ||| | |||||

Sbjct 538416 ACTCTATTGTTTATTTGGTTGTGGCGGCGATCGCGATAAAGGAAAACGACCCCTGATGGC 538475

Query 1230 TGAGATTGCTGAACAGGAAGCGGATCACATCATACTAACCAATGATAACCCGCGCGATGA 1289

| || || ||| | | |||||||| || || |||| |||||||| || || ||||

Sbjct 538476 AGCTATCGCGGAAGAAGGGGCGGATCATATTATTGTAACAAATGATAATCCTCGTTATGA 538535

Query 1290 AGATCCCTTACAAATTTTGCAAGCGATTCAAAAAGGGTTTACAGGTAAAAAATCGGTATA 1349

|||||| || |||||||| |||| |||| | |||| |||| || || ||| || | ||

Sbjct 538536 AGATCCGTTTCAAATTTTTCAAGATATTCGACAAGGTTTTAAGGGAAACAAAGCGATTTA 538595

Query 1350 TCGTGAACCGGATAGACAGCGTGCTATTGCTTATACCTTAGCAACCGCCCAGCCCACTGA 1409

| |||||| || | | || || || ||| | | | |||| || || | ||

Sbjct 538596 TTATGAACCTGAACGGAAAAAAGCGATAGCCTATGCACTGAAAGCCGCTCAACCTTCGGA 538655

Query 1410 TGTGGTTTTAGTGGCGGGTAAAGGACACGAAGCGTACCAGTTGATAAATGGCATTAAGTA 1469

| | |||||| | || || ||||||||||| | || |||| ||| |||| || || ||

Sbjct 538656 TATTGTTTTAATCGCAGGAAAAGGACACGAGTCCTATCAGTCGATTCATGGAATCAACTA 538715

Query 1470 TCCTTTTGATGATGCTATCGAAGTGCAACGTTTTTTAAATCAGTAACTTTTCATTCACTA 1529

||||||||||||||| | ||||| |||| |||||||||||||||| |||| |||

Sbjct 538716 TCCTTTTGATGATGCCGTTGAAGTTCAACATTTTTTAAATCAGTAATTTTTTATT----- 538770

Query 1530 ACCTGACTATCTAAATGTAAATGTATTTATCAGTTTTATCAGTGCAATATCTATAGCTAT 1589

|| ||| ||| |||| || ||| | |||||||||| ||| ||||

Sbjct 538771 ---------TCCAAA-ATAA---GATTT---CGTGTTACCCGTGCAATATCGATAACTAT 538814

Query 1590 CGATTAATATAGCGTTATCtttttttATGGTTAATTTAGTGAAATTATCGACAGTGGCTA 1649

|||||| |||| ||| | |||||| |||||| |||||||||||| || | || |

Sbjct 538815 CGATTACTATA--CTTACC--TTTTTAGGGTTAACGAAGTGAAATTATCAACGCTTGCAA 538870

Query 1650 ACAGACTTCAGGGAAAGCTATTAGGTTCCGATGGCGATTATATCGGACTAAGTTTGGATT 1709

||| || | |||| || | ||| | || | |||| | || |||| ||||||

Sbjct 538871 GCAGGCTGAAAGGAACACTGCTCGGTGCAGACGCTCATTACAAGGGGATAAGCGTGGATT 538930

Query 1710 CGCGTTGCATTAAACCACATGAACTTTTTGTTGCTATACGCGGGGAGCAGTTTGATGGAC 1769

|||||| || ||||| |||||||||||| | || | || || | |||| |||||| |

Sbjct 538931 CGCGTTCTATAAAACCGCATGAACTTTTTATAGCGCTTCGTGGTAAACAGTATGATGGCC 538990

Query 1770 ATCATTTTATTGAACGAGTC-AAACAAAGCGGCGCTGCAGCGGCTATTGTTGATCAGGTT 1828

|||| | | ||| ||| | | || || || || |||||| |||| || ||||

Sbjct 538991 ATCACTATGTTG-GTGAGGCTCAGCAGCATGGTGCAGCGGCGGCTGTTGTGGAAGAGGTG 539049

Query 1829 ATAGAGACTGATTTGCCTTTAGTACTCGTTCAGGATACACGAAAAGCCTTAGGTGAATTA 1888

||||| || ||||| |||||||| ||||| ||||||| || ||||| | |||||| |

Sbjct 539050 ATAGAAACGGATTTACCTTTAGTTCTCGTGGAGGATACGCGGAAAGCGCTCGGTGAACTT 539109

Query 1889 GCTAAGCAACATCGTAGTCAATTTTCTATTCCAGTTATCGCCTTAACGGGTAGCTGTGGA 1948

|| ||||| ||||| |||| ||||||||||| |||| | |||||| || || || ||

Sbjct 539110 GCGAAGCACCATCGGCGTCAGTTTTCTATTCCTATTATTGGCTTAACCGGAAGTTGCGGG 539169

Query 1949 AAAACCACTACCAAAGAAATGATCCGATCTATTTTGGCTGAAGTGGGTCCAGTATTAACT 2008

|||||||| || ||||||||| || ||||||||||| ||| |||| || ||| | ||

Sbjct 539170 AAAACCACAACGCGAGAAATGATTCGTTCTATTTTGGCGGAAATGGGGCCGGTACTGGCT 539229

Query 2009 AATTTTAAAAATTTCAATAATGAGATTGGTTTACCCTTAACCTTGCTGAATTTAAATGCC 2068

|||||||||||||| ||||||| |||||| ||||| ||||| || || |||||| ||

Sbjct 539230 AATTTTAAAAATTTTAATAATGCGATTGGATTACCGTTAACGTTACTTCATTTAACGGCG 539289

Query 2069 GAACACCATTATGCTGTCATCGAAATGGGTGCTAATCATGCGGGAGAAATTGAGTATTTA 2128

|| || | |||||| || || ||||||||||| ||||| || ||||||||||| ||||||

Sbjct 539290 GAGCATCGTTATGCGGTTATTGAAATGGGTGCCAATCACGCCGGAGAAATTGACTATTTA 539349

Query 2129 ACCCAGATTACCCAACCAAATGTTGCGTTGGTCACTAATATTGGACCGGCACATTTGCAA 2188

|| || ||||| ||||| || ||||||| |||| ||||| || || || ||||| |||

Sbjct 539350 ACGCATATTACACAACCTGATATTGCGTTAATCACGAATATAGGTCCTGCTCATTTACAA 539409

Query 2189 GGATTTGGATCGATGGCCGGTATTGCCCAAGCTAAAGCTGAGATTTTTTCTGGTTTAGTT 2248

|||||||| || || | ||||| | |||||||| ||||||||||| || |||| |

Sbjct 539410 GGATTTGGGTCCATTTTGCGCGTTGCCGAGGCTAAAGCAGAGATTTTTTCAGGGTTAGCT 539469

Query 2249 AAGAAGGGTGTCGCTATTATTAATGCGGATGATAAGTTTACTGACGTATTACAAAAAGCC 2308

| ||||| || ||||| |||| || | ||||| || || |||| ||||||||

Sbjct 539470 CAAAAGGGCGTGGCTATCCTTAACGCTGCGGATAATCATAGGGATATATTGGAAAAAGCC 539529

Query 2309 TGTACCGATTTTCGTTGTGTTAGTTTTGGTTTATCCGAGACCTATGAtttttttGCGACT 2368

| | | | ||| |||| | |||||||||| | | | || |||||||| |||| |

Sbjct 539530 AGCGCTCAGTATCGGCGTGTGAATTTTGGTTTAACTCAAAACTGTGATTTTTATGCGCGT 539589

Query 2369 AATATTCAAATAAATACGGACGGGGA-AGCCACGTTTTTATTACATACTCCCTGCAGAAA 2427

| || || | ||| ||||| | | | || |||||||| | || | | || |

Sbjct 539590 CAGATCCAGCTGGATA-AAACGGGAAGACCTACTTTTTTATTGGTTTCT---TCCGGAGA 539645

Query 2428 AGAAATGATGATTCGCTTAAACTTACCTGGACAGCATCAGGTGATGAATGCCTTAGCGGC 2487

||||| || ||||||||||| ||| | ||||| ||||| || |||||||| ||||||||

Sbjct 539646 GGAAATCATCATTCGCTTAAATTTATCGGGACAACATCACGTCATGAATGCATTAGCGGC 539705

Query 2488 TGTGGCCGCGGCAAGTCAAGTGGGCATTGAATTATCGTTTATCAAATCAGGTTTAGAAAA 2547

| ||| | || |||||||||| | ||||| || |||| |||| || ||||||||

Sbjct 539706 CGCGGCGGTCGCGCGTCAAGTGGGTGTGGAATTCTCTGTTATTCAATCCGGCTTAGAAAA 539765

Query 2548 AGCTCAGCCGGTACCAGGAAGAGCGATAGTGAGTACAACAAAAATAGGTGTGACACTCAT 2607

| || || || | || |||| ||| | |||||||||| ||| | |||| | ||

Sbjct 539766 AATGCAACCCTTATCCGGGAGAGTGATCAATCGCACAACAAAAACAGGCGCGACATTGAT 539825

Query 2608 AGATGATAGTTATAATGCCAATCCGAGCTCAGTCGCCGTGGCCTTGAAATTATTAGCCCA 2667

||||| || |||||||| |||||||| ||||| || | |||||| ||||| ||||| ||

Sbjct 539826 TGATGACAGCTATAATGCGAATCCGAGTTCAGTGGCGGCGGCCTTAAAATTGTTAGCACA 539885

Query 2668 TTACACAGGCCTACGAATTTTTGTTATGGGTGATATGGGTGAGTTAGGTAAAAATGCGGT 2727

||||| |||||| |||||||| ||| ||||||||||| ||||||| ||||||| |||||

Sbjct 539886 TTACAAAGGCCTGCGAATTTTGGTTCTGGGTGATATGAGTGAGTTGGGTAAAAGTGCGGG 539945

Query 2728 GGACTATCATCGTCAAATCGGAGAATTAGCCAAAGAATTAAACATCGATGAGGTCTATAC 2787

| ||||||||||||||| ||||||||||| |||| |||| |||||| | ||||||||

Sbjct 539946 TGCCTATCATCGTCAAATGGGAGAATTAGCAAAAGCGTTAACGATCGATAAAGTCTATAC 540005

Query 2788 TTACGGTGATTTAAGTAAAGAAACGGCTAAGGCATTTGGTGCCAATGGAAAGCATTATTC 2847

|| |||||||||||| ||||||| || || || ||||||||| |||| || || |||||

Sbjct 540006 TTGTGGTGATTTAAGTGAAGAAACAGCCAAAGCGTTTGGTGCCCATGGCAAACACTATTC 540065

Query 2848 CAATCATCAAAAATTAATTTTAGCTCTAAAATTGCGTTTGCAAGAGAAGGTGACCATTTT 2907

|||||||||||||| |||||||| ||||| | | ||| | || ||||| |||||

Sbjct 540066 AAATCATCAAAAATTGATTTTAGCGTTAAAACCCGATGTACAAAAAAACGTGACTATTTT 540125

Query 2908 AATTAAAGGTTCGCGTACTGCGCAAATGGAAAAAGTAGCCCGTGCTTTAATGGATTAATA 2967

|||||||||||| || | ||| |||||||| |||||||| ||||||||||||||||||

Sbjct 540126 AATTAAAGGTTCACGCAATGCACAAATGGAGAAAGTAGCGGCTGCTTTAATGGATTAATA 540185

Query 2968 ATGTGTGAACAATAAC 2983

|| |||| | |||||

Sbjct 540186 ATATGTGGATGATAAC 540201

**Supplementary Figure 4.** Read alignments do not support assembly errors predicted by REAPR in *Rickettsiella isopodorum* candidate genomic islands. Predicted assembly errors do have lower coverage than other regions, but raw sequence reads still align to and span these regions, and there is no evidence of discordantly mapped reads. The plots below were generated by IGV. Only the candidate genomic islands that overlapped with predicted assembly errors are shown below.

A. Contig 191:


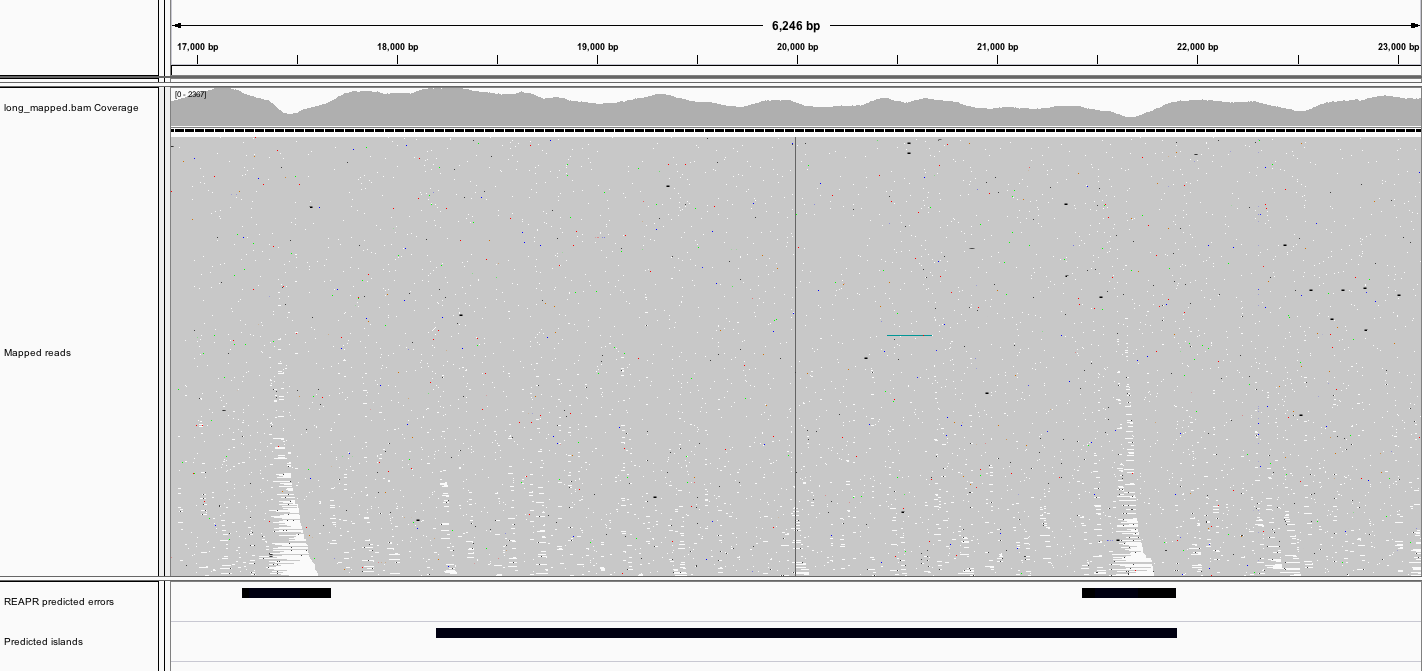


B. Contig 193:
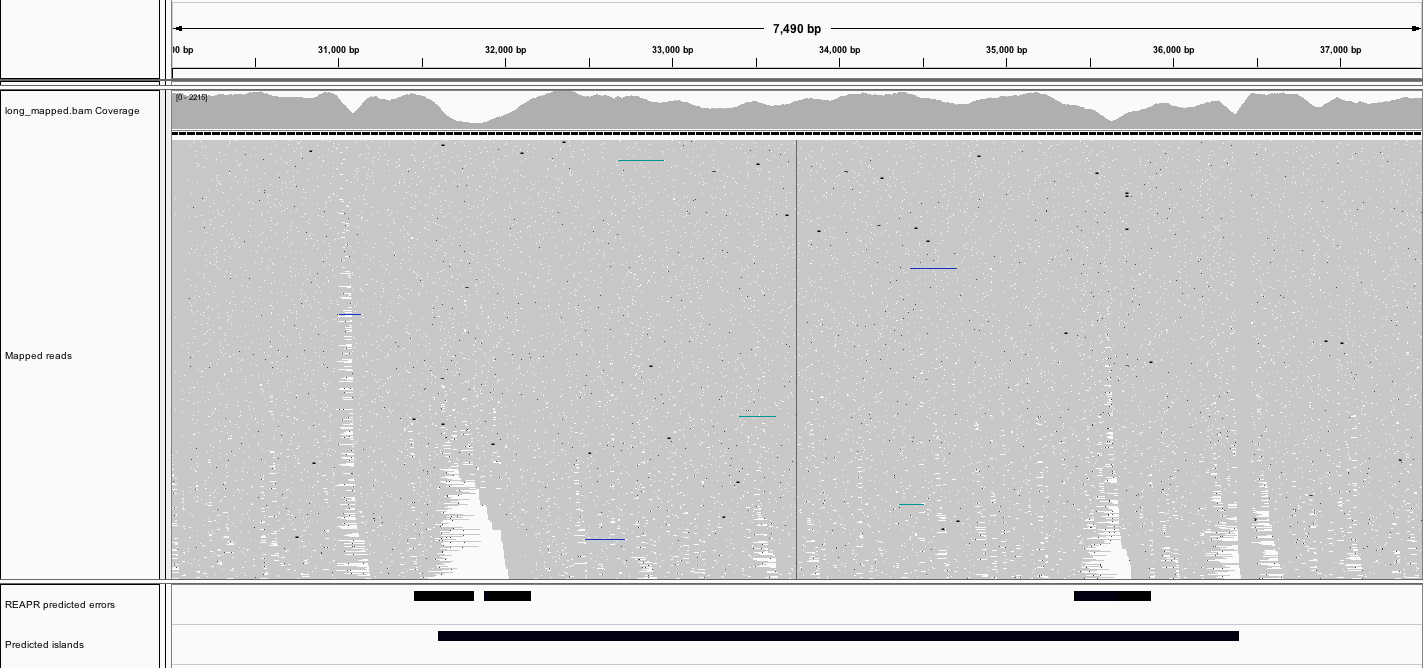


C. Contig 197:


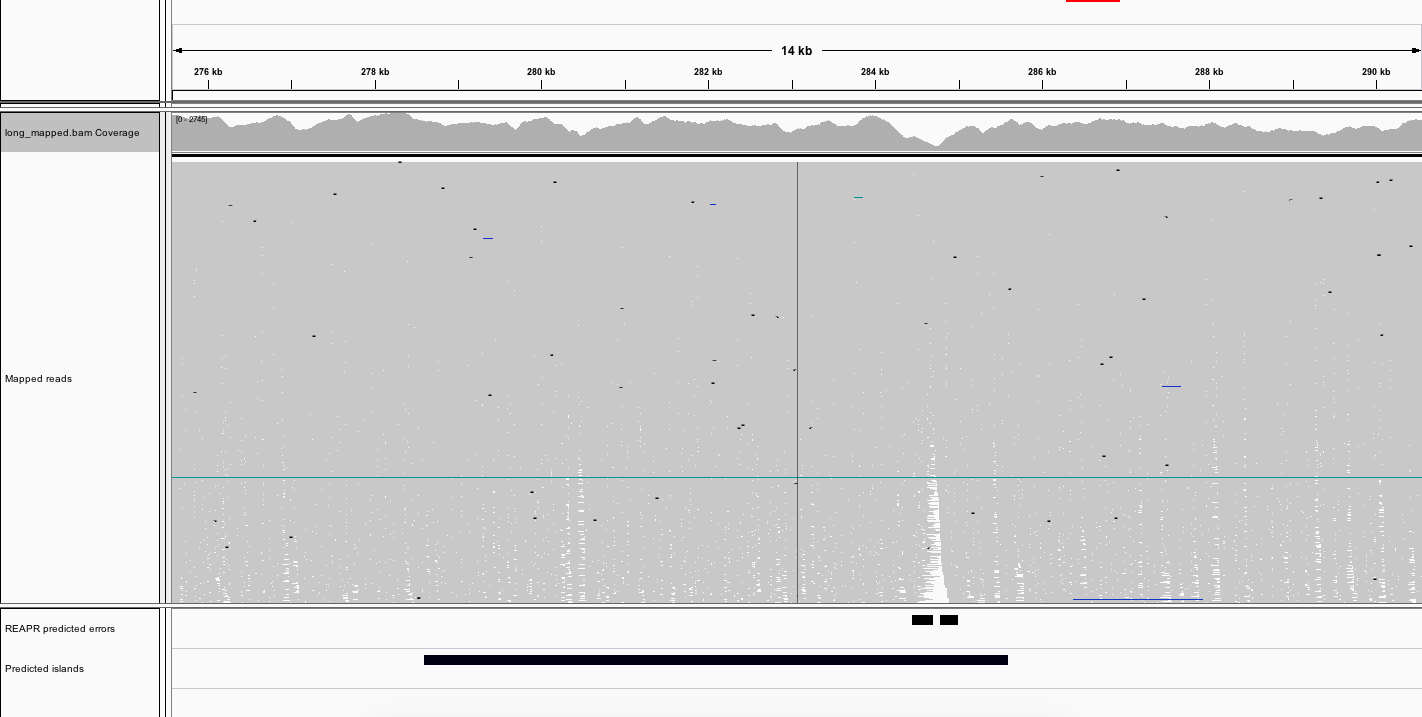


D. Contig 197 (second candidate island):


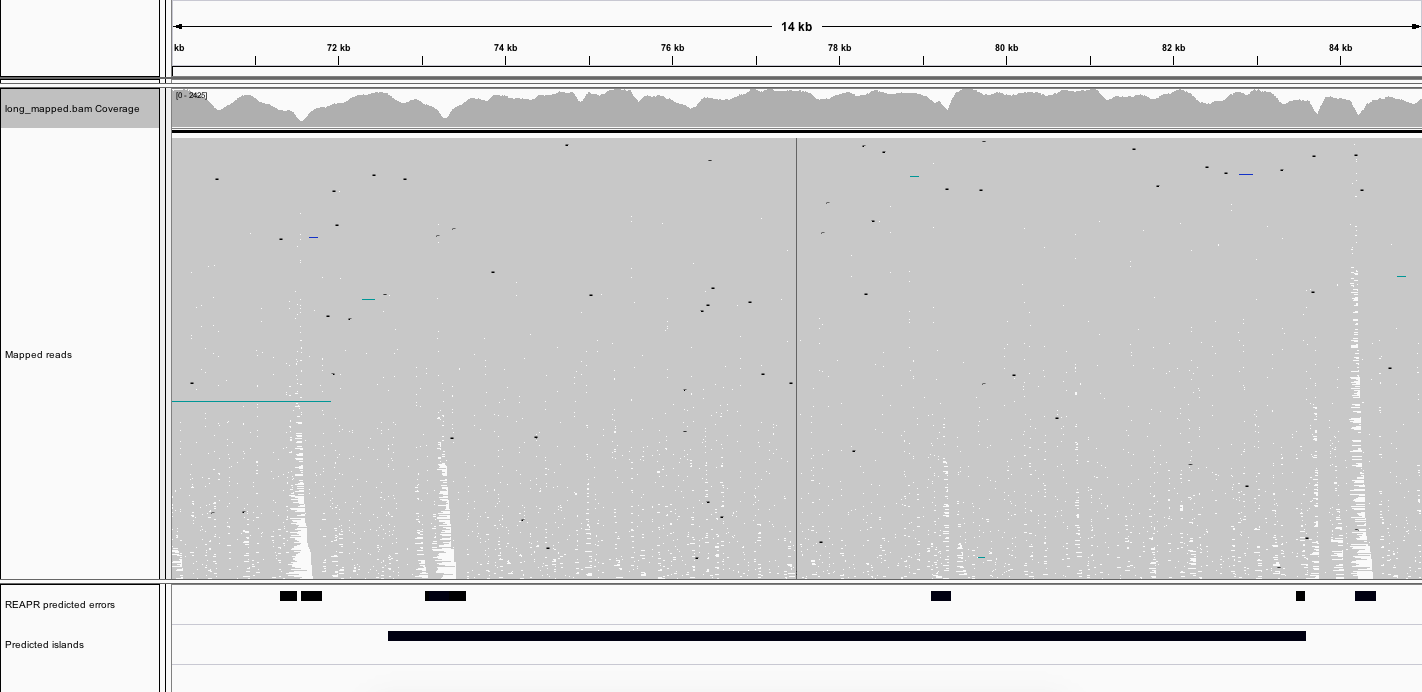


E. Contig 197 (third candidate island):


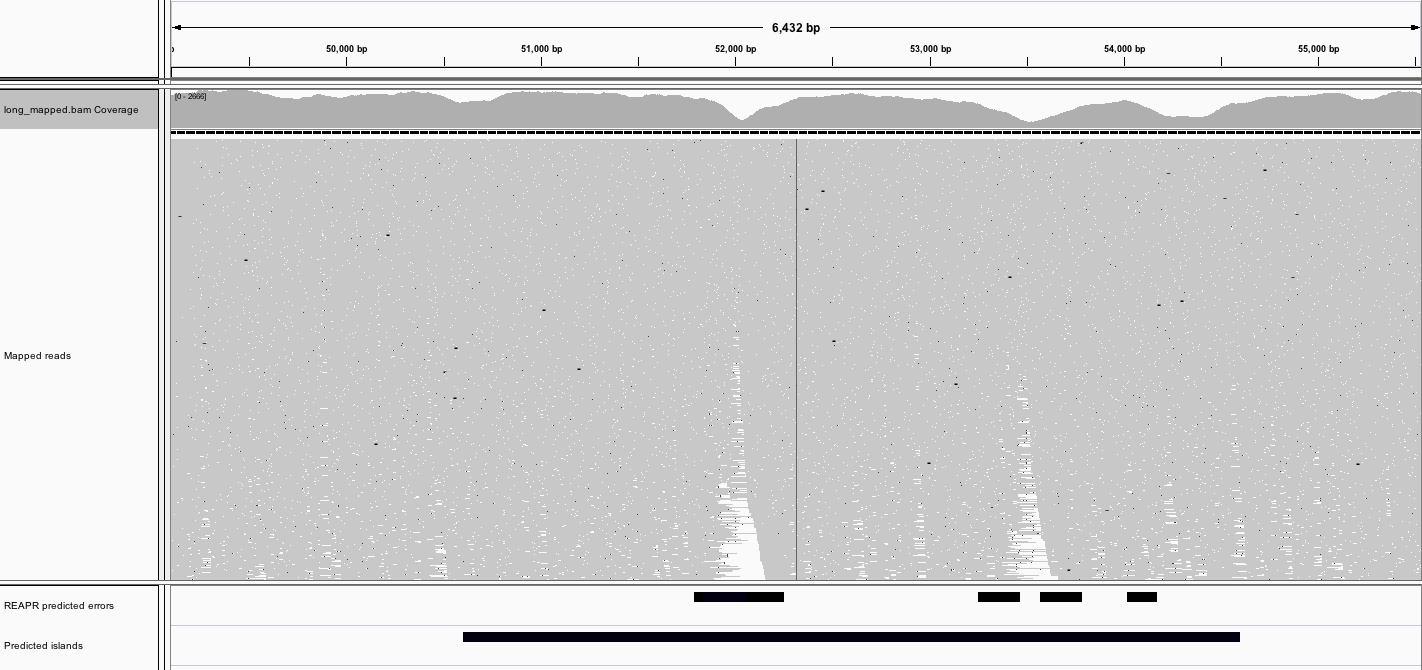


**Supplementary Figure 5.** Blobplot created from an assembly of all sequence data from the female *T. rathkei* isopod sample. Although multiple clusters of bacterial contigs are present, they are easily separated from the *R. isopodorum* contigs because of their higher GC content and lower coverage.


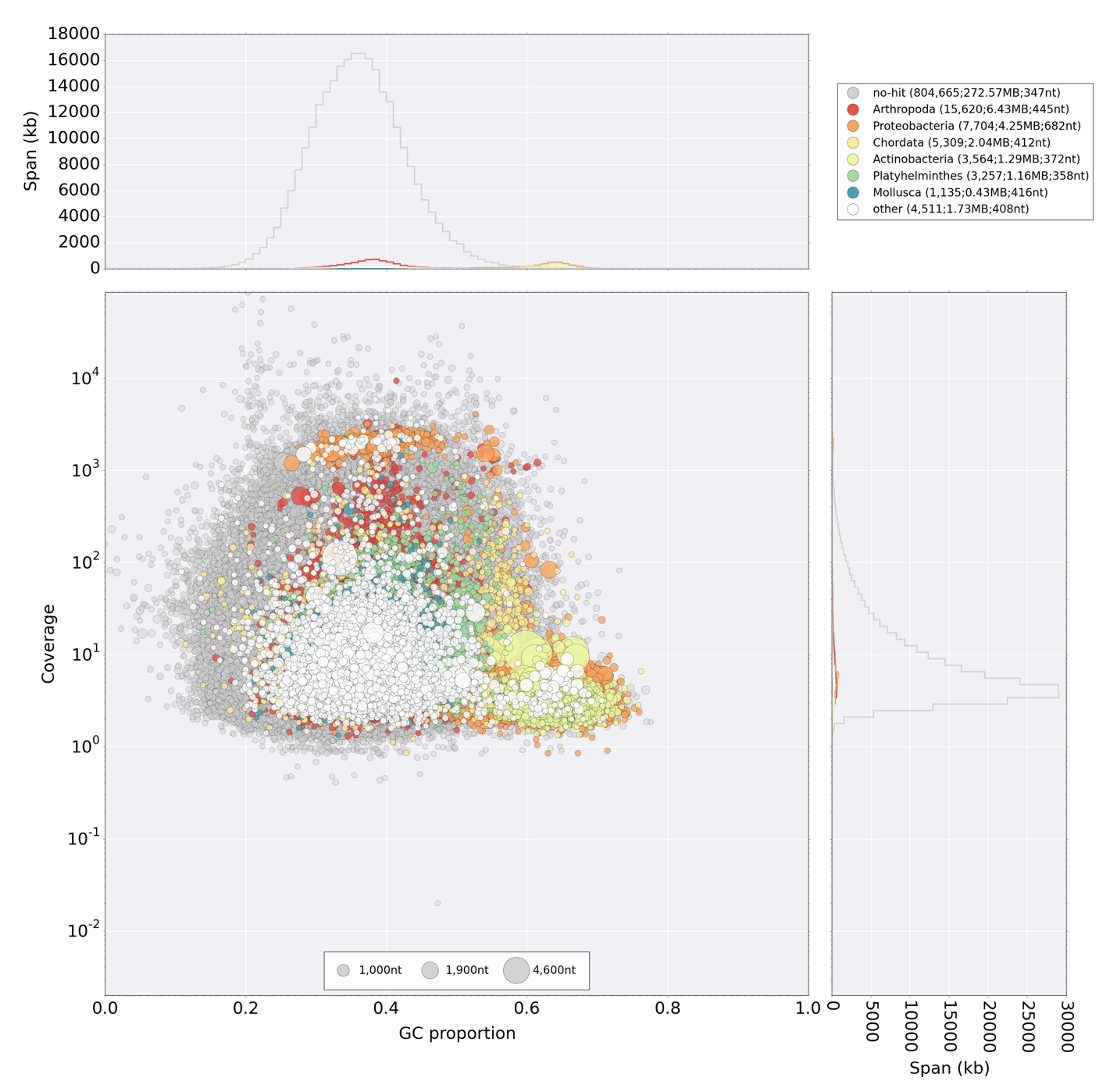


**Supplementary Figure 6.** Blobplot created from our final *R. isopodorum* assembly. There is one relatively short contig with higher coverage and higher GC content, but it is unlikely to be a contaminant because this sequence contains matches to 16S and 23S rRNA sequences from other *Rickettsiella* isolates. There are a few other contigs with no BLAST hits, but these are all short (total length of only 0.02 Mb) and have relatively high coverage, suggesting that these also represent multi-copy-number sequences. Thus the remaining fragmentation in our final assembly is likely caused by repeat sequences.


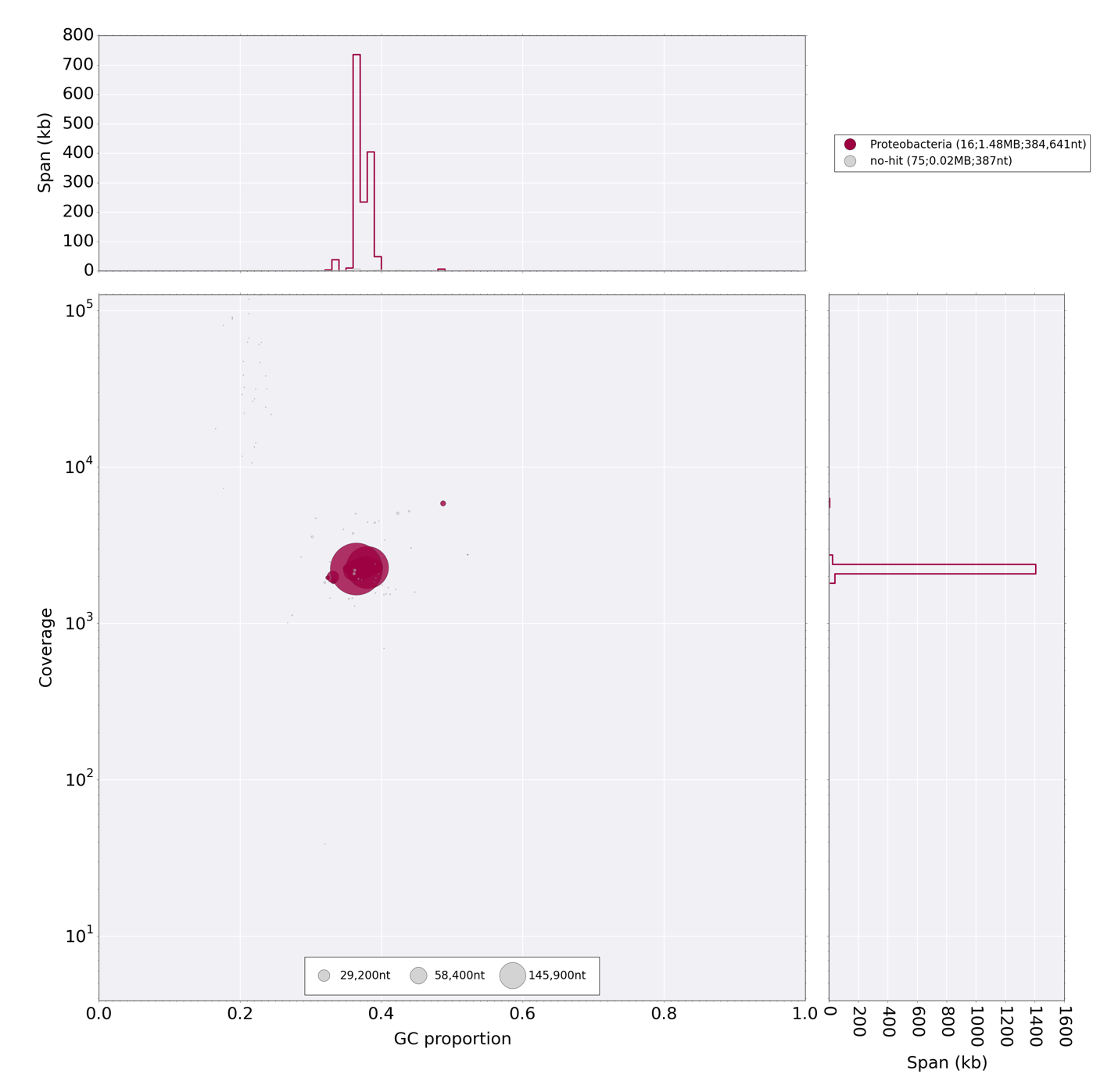


**Supplementary Figure 7.** Several short contigs in our *R. isopodorum* assembly match multiple locations in the previously sequenced *R. grylli* genome, suggesting that the short, fragmented contigs in our final assembly could not be better assembled because they are repeat sequences.

Query= contig_134

Length=127

Score E

Sequences producing significant alignments: (Bits) Value

NZ_AAQJ02000001.1 Rickettsiella grylli gcontig_637, whole genom... 170 6e-44

> NZ_AAQJ02000001.1 Rickettsiella grylli gcontig_637, whole genome

shotgun sequence

Length=1566411

Score = 170 bits (188), Expect = 6e-44

Identities = 114/127 (90%), Gaps = 0/127 (0%)

Strand=Plus/Plus

Query 1 TACTCAGTATTTTATTCAATGAAAATGTATCTTCCGTGGGCAAACGACAATTTTTAGGTA 60

||||||| || ||||||||||||||||||||| ||||||||||| || ||||||||||||

Sbjct 430142 TACTCAGCATCTTATTCAATGAAAATGTATCTGCCGTGGGCAAAAGAAAATTTTTAGGTA 430201

Query 61 AAGCACTGCAAGTGGCTTCGCCATTTGTTGCTAAAGCCACCTCAATTTATTTTGCCTATA 120

||| ||||| |||||||||||||||||| ||||| ||||||||||||||||| ||||

Sbjct 430202 AAGTGATGCAATCGGCTTCGCCATTTGTTGCGAAAGCTACCTCAATTTATTTTGCTTATA 430261

Query 121 ATCTTAA 127

|||||||

Sbjct 430262 ATCTTAA 430268

Score = 116 bits (128), Expect = 1e-27

Identities = 73/79 (92%), Gaps = 0/79 (0%)

Strand=Plus/Minus

Query 49 AATTTTTAGGTAAAGCACTGCAAGTGGCTTCGCCATTTGTTGCTAAAGCCACCTCAATTT 108

|||||||||| |||||| ||||| |||||||||| |||||||| ||||||||||||||||

Sbjct 201504 AATTTTTAGGCAAAGCAATGCAAATGGCTTCGCCTTTTGTTGCGAAAGCCACCTCAATTT 201445

Query 109 ATTTTGCCTATAATCTTAA 127

||||||| |||||||||||

Sbjct 201444 ATTTTGCTTATAATCTTAA 201426

----

Query= contig_147

Length=157

Score E

Sequences producing significant alignments: (Bits) Value

NZ_AAQJ02000001.1 Rickettsiella grylli gcontig_637, whole genom... 223 1e-59

> NZ_AAQJ02000001.1 Rickettsiella grylli gcontig_637, whole genome

shotgun sequence

Length=1566411

Score = 223 bits (246), Expect = 1e-59

Identities = 131/136 (96%), Gaps = 0/136 (0%)

Strand=Plus/Plus

Query 18 TGGCGCAAAATGAATGCCTGGCGACCTTAGCGAGTAGGTCCCACCTGATCCCATTCCGAA 77

||||||||||||||||||||||||||||||||| ||||| ||||||||||||||||||||

Sbjct 277049 TGGCGCAAAATGAATGCCTGGCGACCTTAGCGAATAGGTACCACCTGATCCCATTCCGAA 277108

Query 78 CTCAGACGTGAAACTACTCTGCGCCAATGATACTTTGGGGTTCCCCCATGGGAAAGTAGG 137

||||| |||||||||| |||||||||||||||||||||||||||||||||||||||||||

Sbjct 277109 CTCAGTCGTGAAACTATTCTGCGCCAATGATACTTTGGGGTTCCCCCATGGGAAAGTAGG 277168

Query 138 GCATCGCCAGGCTACT 153

|||| |||||||||||

Sbjct 277169 GCATTGCCAGGCTACT 277184

Score = 223 bits (246), Expect = 1e-59

Identities = 131/136 (96%), Gaps = 0/136 (0%)

Strand=Plus/Plus

Query 18 TGGCGCAAAATGAATGCCTGGCGACCTTAGCGAGTAGGTCCCACCTGATCCCATTCCGAA 77

||||||||||||||||||||||||||||||||| ||||| ||||||||||||||||||||

Sbjct 646855 TGGCGCAAAATGAATGCCTGGCGACCTTAGCGAATAGGTACCACCTGATCCCATTCCGAA 646914

Query 78 CTCAGACGTGAAACTACTCTGCGCCAATGATACTTTGGGGTTCCCCCATGGGAAAGTAGG 137

||||| |||||||||| |||||||||||||||||||||||||||||||||||||||||||

Sbjct 646915 CTCAGTCGTGAAACTATTCTGCGCCAATGATACTTTGGGGTTCCCCCATGGGAAAGTAGG 646974

Query 138 GCATCGCCAGGCTACT 153

|||| |||||||||||

Sbjct 646975 GCATTGCCAGGCTACT 646990

----

Query= contig_168

Length=254

Score E

Sequences producing significant alignments: (Bits) Value

NZ_AAQJ02000001.1 Rickettsiella grylli gcontig_637, whole genom... 309 2e-85

> NZ_AAQJ02000001.1 Rickettsiella grylli gcontig_637, whole genome

shotgun sequence

Length=1566411

Score = 309 bits (342), Expect = 2e-85

Identities = 221/254 (87%), Gaps = 0/254 (0%)

Strand=Plus/Plus

Query 1 TAGAAGAGGATACTGAAATTCAACTATTGAAAAACATTGGAAATTATGCATTCTTTCGTG 60

|||||| ||||||||||||||| ||||||||||||||||||| |||| || |||||||

Sbjct 432386 TAGAAGTGGATACTGAAATTCAGATATTGAAAAACATTGGAAACTATGACTTTTTTCGTG 432445

Query 61 ATGAAACTAATCTTATACTTACTAATGCCTTCGAAGTTTCAAATGATTACTGCACAATTA 120

||||||| |||||||||| || |||||||||||||||||||||||||||||||| ||||

Sbjct 432446 ATGAAACCCATCTTATACTGACGAATGCCTTCGAAGTTTCAAATGATTACTGCACGATTA 432505

Query 121 TCTGCCGCCATTTTTATCAAGAACCGGAAATGAGAAAAAAGATATTATCGACGACATTTA 180

|||| | | |||||||||||||| | |||||||||||||| ||||||| | ||||

Sbjct 432506 TCTGTCACAATTTTTATCAAGAATCAGAAATGAGAAAAAAAGTATTATCAGTCATTTTTA 432565

Query 181 AATTTTTTGACCAAGAAATTCATCCTAATGACCATCAAAAACAAATTGAGCATGCGACAC 240

|||||| ||||||||||||||||||||| ||| ||||| ||||||| |||||||| | |

Sbjct 432566 AATTTTATGACCAAGAAATTCATCCTAAGGACTATCAAGAACAAATCGAGCATGCTGCGC 432625

Query 241 ATTTTCAACATTTA 254

||||||||||||||

Sbjct 432626 ATTTTCAACATTTA 432639

Score = 69.8 bits (76), Expect = 4e-13

Identities = 144/212 (68%), Gaps = 2/212 (1%)

Strand=Plus/Minus

Query 25 TATTGAAAAACATTGGAAATTATGCATTCTTTCGTGATGAAACTAATCTTATACTTACTA 84

|||||||||||| |||| | | | | || ||||| ||| || | ||||| | || |

Sbjct 199287 TATTGAAAAACAGTGGAGACTTTTCTTTTTTTCGCCATGCGACAGACCTTATCTTAACGA 199228

Query 85 ATGCCTTCGAAGT-TTCAAATGATTACTGCACAATTATCTGCCGCCATTTTTATCAAGAA 143

|| || | || || | |||||| || ||||| |||| || || ||||| ||

Sbjct 199227 AT-ATTTTTACGTCCTCTATTGATTATTGGACAATAATCTATTATCAGTTCTATCAGGAC 199169

Query 144 CCGGAAATGAGAAAAAAGATATTATCGACGACATTTAAATTTTTTGACCAAGAAATTCAT 203

|| ||||| || ||| |||||| | ||||||||| || | ||||||||| |

Sbjct 199168 AAGGCAATGAAAAGAAAAGCATTATCAGCCTATTTTAAATTTCTTAATCAAGAAATTTGT 199109

Query 204 CCTAATGACCATCAAAAACAAATTGAGCATGC 235

| || | |||||||| ||||||| || ||

Sbjct 199108 CTAAAAAATTATCAAAAAGAAATTGAACACGC 199077

**Supplementary Figure 8.** Insertion sequences predicted by ISsaga. Sequence names indicate: contig, start position, end position, length, insertion sequence classification.

>Contig197_18389_19576_1188_ISL3

gtgggaatattatacggtgtattaaatttatctttttggggatatgtcgttgccttattaattttaacgcatatcactattgtgggtgtcacggtgtatttgcaccgatctcaagcgcatcgtgctttagagctgcatcccgccattagtcatttcttccgtttttggatctggttgaccacgggtatggaaaccaaaaaatgggtttctattcaccgtaaacatcatgctaagtgtgaaacggacgaagatccacatagtccgcaaactcgaggcattaaaaaagttttttttgagggtgctgagttgtatcgtgacgaagctaaaaatcaagatacgatggaccgttatggtcaagggacaccagatgactggttagaacgacatgtctatactaagcatagcgcggcaggtattggattgatgtttgttattgatctgattttatttggtattcctgggattaccatttgggctttacagatggcttggatacctttctttgccgcgggtgtggtgaatggtatcggccactattggggctatcgaaactttgaatgtcctgatgcggcgcgaaatattattcctttaggcgcctttattggtggtgaggaattgcataataatcatcatacctttccaacttcagctaagttctcggtcaaatggtgggaatttgatttgggttgggtttatattcgcttattacaatttttaggtttatccaaggtaaaacgagtatcacccaaacttgagaatattccaggaaaatcactgattgattcagatactttagccgcattgattacgaatcgttttcaagtgttggcgcgctatagtcgtgaggtgttattaccggtcttgcatgaagaaaagctaaaagcgaatactagcagtaaagcgttattaaagcgggctaagatcgcattaattcgtactgagtctttattaaacgaagaaggtaaacagcaaatagctgaagtgatagataatcatcatatgttagcgttagtttatcagtaccgtcttaaactacaggcgatttggggccgtaccacagcaacgcagcgcgagttattagaagccttacaggattggtgtaaacaagccgaggcaacgggtgtgcatgcgttacggaaatttgcgataagtttagccggattttcgacgcaaaaaaaattaacttaa

>Contig190_4886_5077_192_ISNCY_ssgr_ISPlu15

aaatcagccatagaaaaagggcttcaacaagggctacagcaagggctacagcaagggctacagcaaggccgtgaagaaggtcgtgaagaaggtgaataccttaaagctactattatagctaaaaagcttatagctcaaggtagatcaattcaatacattcaagatcttaccaatttatctgaaaatgaaata

**Supplementary Figure 9.** Components of predicted prophage element on contig 198.

| **Location** | **BLAST hit** | **E-value** | **Amino acid sequence** |
| --- | --- | --- | --- |
| complement(116125..116919) | PHAGE_Deep_s_D6E_NC_019544: thymidylate synthase; PP_00920; phage(gi423262375) | 3.46E-132 | MRPYLDFLQHILNQGQAKADRTGTGTLSIFAYQMRFDLSVGFPLVTTKKLHLKSIIYELLWFLRGDTNIRYLNEHQVTIWDEWADSAGNLGPIYGKQWRSWLTTDQKIIDQISQLIEQIKANPDSRRLIVSAWNVGELAKMALPPCHLLFQFYVANSRLSCQLYQRSADAFLGVPFNIASYALLTHMIAQQCNLQVGEFIWTGGDCHIYSNHLEQVNCQLSRQPYRVPQLNILRKPKTLFDYVFDDFSLVDYKCHSLIKAAVAV |
| 117077..118408 | PHAGE_Synech_S_SSM7_NC_015287: hypothetical protein; PP_00921; phage(gi326783917) | 5.80E-30 | MRINLFGAGYVGLVTATCLAEHGNKVLCIDIDQEKVKRLQQGECPIHEPDLPALLQKNLSAGRLNFSTDPQQGVEHGFYQFITVGTPQDEDGSADLTYVLKVAEYIGKTLLEPKLIINKSTVPVGTADKVKAIIQNQLHQRNINIPFNIASNPEFLREGVAVNDFMRSDRIIIGTDNNDAESHLRHLYRPFNRNNDRLIAMDIRSAELTKYAANAFLATKISFINEMSHLAERLNADIEQVRIGIGSDPRIGYHFINPGCGYGGSCFPKDVIALEATAKTVHYQPQLLNAVHQVNDAQKKLLFSKVSQFFQNNLRGKVVALWGLSFKPNTDDMREAPSKVFIAAALAAGMRIQAYDPVAMPEAVRLYKDQVNFSCCDNPEDSLVGADVLVIVTEWNIFFNPDFQLIKQRLKYPAIFDGRNLYDPDCLKQLGIKYYAIGRGEPL |
| 118410..119351 | PHAGE_Sphing_PAU_NC_019521: gp187; PP_00922; phage(gi435844690) | 4.21E-14 | MKKIKKITKAIFPVAGLGTRFLPATKASPKEMLPIVDKPLIQYAVEEAIAAGITELIFITSSSKRAIEDHFDSNYELEAKLAEAGKKDLLAIVKNILPKGVSCVYLRQPDTLGLGHAVLCAQTLINDEAFAVLLADDLIDSTLPCLKQMLDLYQEKQNTIIAVQAITPEESKQYGIIGYKTKEGKLSQINAIVEKPSHKEAPSNLAVVGRYILTSTIFFYLSKTPIGKNGEIQLTDAIAHQLKDEVIYAWEFEGTRYDCGSKFGYLKATIAHALKHPETKNLFIHYLKTLRNNFHEDCELNGNTDKNSSTKRI |
| complement(119398..120723) | PHAGE_Salmon_SEN34_NC_028699: tail tape measure protein; PP_00923; phage(gi966201433) | 2.92E-08 | MTYKMLKIYSLLLTVLLILAVGITACVQFPTSEEGVKGLSGSGNDTDRKTYQTVIMKSTGSSLAPQNAKAMYSAVEKGTLWGPIRAHFQLSAREENQPQVQKQIRWFARNPLYLKDAVNRAAPYIYYVYAQVRKRDLPTELVLLPIIESGYNPSATNSSSGAAGLWQLMANTAKGYGVHQNRGFDGRRDISSSTNAALNYLTYLRSFFGGDWLLAIAAYDTGEGNVQNAIRHNTEQDKNTHFWALPLASETRSYIPRLLALAAIVKNPAKYGVSLPPVSAKPYLELVDAKNMSLTHVAKLAGMNVSELKELNPGVKSTSAAIKRGQLALPIDRVALYKQQLAAASSPNFKVQVGNNKLALKQARSKGKQRSVQLVNNEQAKPRSATLVKDKSSSQIYWVKSGDTLTGIAKHYHISVKKIQAWNKLDSDFLKPGEKLKIMLS |
| 120997..121719 | hypothetical; PP_00924 | N/A | LKQHYLDIENHRLANLIPHYAGRHLLQLSPYSFSSLSTSPIIHKIIISSNYKCKHITDKINSSHLESHYTHLPFANDSINLVLMPHTLEVNKSTAQTILTEAWRVLAPSGHLIILGINPISLWGLYRLFSLSKKPTWGDGRFHTIQTLCQWIHFLGGEIQHTESFLFRPPLSSPPGMWLFKKLVWLERVSPWLIPFMGGIYLIIAEKRVKRLNGLGLVWQFPPVLNHKVLAPNARGPHHA |
| 121856..122158 | PHAGE_Salmon_SSU5_NC_018843: putative ribonuclease H; PP_00925; phage(gi410491500) | 5.89E-32 | MELMAAIQALMAIKKPCHITLSTDSQYVQKGITEWLPQWKRRAWLTANKKPVKNSDLWKKLAIQAERHQISWEWVKGHSGHPENDRVDSLANAALDELLE |
| complement(122195..123145) | hypothetical; PP_00926 | N/A | VSRKNNSIQTSRALKQRTLKNVIKAAGITLHSGETAILTLRPAPINTGIIFRRLDFNPIVEVQARAEHVGETTLQTTLLKNGVRVATIEHLMSAMAGLGIDNAYVDITASEIPIMDGSAGPFIFLIQSAGIEEQTASKRFIRIKQSIKVTEGDKWASFEPFDGFKVSFEIDFNHPLFQNRSQKASIDFSTTSYIKEVSRARTFGFMADYEKLREVRLALGGSLDNAVVVDEYRVLNEDGLRYEDEFVRHKILDAVGDLYLLGHSLIGAFSGYKSGHALNNLLLRRLLSNANAWEYVEFEDEIKAPLAYRRALLNAH |
| complement(123148..124323) | PHAGE_Bacill_G_NC_023719: gp138; PP_00927; phage(gi593777597) | 1.98E-09 | MSTEFDSPNTPVQNAIIKVIGVGGGGGNALEHMLAQDITGVEFICANTDAQALRNSSAECLLQLGQQITKGLGAGADPEVGRLAAEADRERIRSALEGANMVFITAGMGGGTGTGAAPVVAEIAKQMKILTVAVVTKPFEIEGKKRLRLAEEGIKQLSQYVDSLITIPNNKLMSVLDKDISFLDAFKAVNDVLFGAVKGIAALITRTGLINVDFADVKTVMSEMGMAMMGTGIGTGPERARLAAEAAIGSPLLEDIDLAGARGVLVNITAGPDLSMREFGEVGEVIKKFTSEEANVVIGTVIDSEMCEELRVTIVITGLLGHLSTGVSGGVGDSSLVRAADGSLDYHQLERPTVLRKQGVVTSSKSTVDSSATDIEYFDIPAFLRRQEEVS |
