# Supplementary material for: Candidate pathogenicity islands in the genome of ‘Candidatus Rickettsiella isopodorum’, an intracellular bacterium infecting terrestrial isopod crustaceans
Source: PeerJ. 2016 Dec 21;4:e2806. doi: 10.7717/peerj.2806 (PMC5181103; doi:10.7717/peerj.2806)
Supplement: Supplemental Information 2 [file peerj-04-2806-s002.zip › Supplement/blastkoala_results/BlastKOALA_Result_MODULE.pdf]

Module Reconstruction Result

Show all objects

Pathway module

Energy metabolism

Carbon fixation

M00166 Reductive pentose phosphate cycle, ribulose-5P => glyceraldehyde-3P [PATH:map01200 map00710] (2) (2 blocks missing)
M00167 Reductive pentose phosphate cycle, glyceraldehyde-3P => ribulose-5P [PATH:map01200 map00710] (5) (2 blocks missing)
M00168 CAM (Crassulacean acid metabolism), dark [PATH:map01200 map00710] (1) (1 block missing)

Methane metabolism

M00345 Formaldehyde assimilation, ribulose monophosphate pathway [PATH:map01200 map00680] (1) (2 blocks missing)

Carbohydrate and lipid metabolism

Central carbohydrate metabolism

M00001 Glycolysis (Embden-Meyerhof pathway), glucose => pyruvate [PATH:map01200 map00010] (10) (1 block missing)
M00002 Glycolysis, core module involving three-carbon compounds [PATH:map01200 map01230 map00010] (7) (complete)
M00003 Gluconeogenesis, oxaloacetate => fructose-6P [PATH:map00010 map00020] (8) (1 block missing)
M00307 Pyruvate oxidation, pyruvate => acetyl-CoA [PATH:map01200 map00010 map00020 map00620] (6) (complete)
M00009 Citrate cycle (TCA cycle, Krebs cycle) [PATH:map01200 map00020] (15) (complete)
M00010 Citrate cycle, first carbon oxidation, oxaloacetate => 2-oxoglutarate [PATH:map01200 map01210 map01230 map00020] (4) (complete)
M00011 Citrate cycle, second carbon oxidation, 2-oxoglutarate => oxaloacetate [PATH:map01200 map00020] (11) (complete)
M00007 Pentose phosphate pathway, non-oxidative phase, fructose 6P => ribose 5P [PATH:map01200 map01230 map00030] (3) (1 block missing)
M00580 Pentose phosphate pathway, archaea, fructose 6P => ribose 5P [PATH:map01200 map01230 map00030] (1) (1 block missing)
M00005 PRPP biosynthesis, ribose 5P => PRPP [PATH:map01200 map01230 map00030 map00230] (1) (complete)
M00308 Semi-phosphorylative Entner-Doudoroff pathway, gluconate => glycerate-3P [PATH:map01200 map00030] (4) (1 block missing)

Other carbohydrate metabolism

M00012 Glyoxylate cycle [PATH:map01200 map00630] (4) (2 blocks missing)
M00632 Galactose degradation, Leloir pathway, galactose => alpha-D-glucose-1P [PATH:map00052] (2) (2 blocks missing)
M00061 D-Glucuronate degradation [PATH:map00040 map00030] (3) (2 blocks missing)
M00549 Nucleotide sugar biosynthesis, glucose => UDP-glucose [PATH:map00520] (1) (2 blocks missing)
M00793 dTDP-L-rhamnose biosynthesis [PATH:map00521 map00523] (4) (complete)

Fatty acid metabolism

M00082 Fatty acid biosynthesis, initiation [PATH:map01212 map00061] (6) (complete)
M00083 Fatty acid biosynthesis, elongation [PATH:map01212 map00061] (5) (complete)
M00086 beta-Oxidation, acyl-CoA synthesis [PATH:map01212 map00071] (1) (complete)
M00087 beta-Oxidation [PATH:map01212 map00071] (2) (1 block missing)

Lipid metabolism

M00093 Phosphatidylethanolamine (PE) biosynthesis, PA => PS => PE [PATH:map00564] (3) (complete)

Lipopolysaccharide metabolism

M00060 Lipopolysaccharide biosynthesis, KDO2-lipid A [PATH:map00540] (8) (1 block missing)
M00063 CMP-KDO biosynthesis [PATH:map00540] (3) (1 block missing)

M00064 ADP-L-glycero-D-manno-heptose biosynthesis [PATH:map00540] (4) (complete)
Terpenoid backbone biosynthesis
M00096 C5 isoprenoid biosynthesis, non-mevalonate pathway [PATH:map00900] (7) (1 block missing)
M00364 C10-C20 isoprenoid biosynthesis, bacteria [PATH:map00900] (1) (1 block missing)

Nucleotide and amino acid metabolism

Purine metabolism
M00049 Adenine ribonucleotide biosynthesis, IMP => ADP,ATP [PATH:map00230] (5) (complete)
M00050 Guanine ribonucleotide biosynthesis IMP => GDP,GTP [PATH:map00230] (5) (complete)
Pyrimidine metabolism
M00051 Uridine monophosphate biosynthesis, glutamine (+ PRPP) => UMP [PATH:map00240] (8) (1 block missing)
M00052 Pyrimidine ribonucleotide biosynthesis, UMP => UDP/UTP,CDP/CTP [PATH:map00240] (3) (complete)
M00053 Pyrimidine deoxyribonuleotide biosynthesis, CDP/CTP => dCDP/dCTP,dTDP/dTTP [PATH:map00240] (8) (1 block missing)
Cysteine and methionine metabolism
M00338 Cysteine biosynthesis, homocysteine + serine => cysteine [PATH:map01230 map00270] (2) (complete)
M00035 Methionine degradation [PATH:map00270] (3) (1 block missing)
M00368 Ethylene biosynthesis, methionine => ethylene [PATH:map00270] (1) (2 blocks missing)
Lysine metabolism
M00016 Lysine biosynthesis, succinyl-DAP pathway, aspartate => lysine [PATH:map01230 map00300] (8) (1 block missing)
M00526 Lysine biosynthesis, DAP dehydrogenase pathway, aspartate => lysine [PATH:map01230 map00300] (5) (1 block missing)
M00527 Lysine biosynthesis, DAP aminotransferase pathway, aspartate => lysine [PATH:map01230 map00300] (6) (1 block missing)
Arginine and proline metabolism
M00015 Proline biosynthesis, glutamate => proline [PATH:map01230 map00330] (1) (1 block missing)
Other amino acid metabolism
M00027 GABA (gamma-Aminobutyrate) shunt [PATH:map00250] (1) (2 blocks missing)
Cofactor and vitamin biosynthesis
M00127 Thiamine biosynthesis, AIR => thiamine-P/thiamine-2P [PATH:map00730] (4) (1 block missing)
M00125 Riboflavin biosynthesis, GTP => riboflavin/FMN/FAD [PATH:map00740] (6) (complete)
M00120 Coenzyme A biosynthesis, pantothenate => CoA [PATH:map00770] (4) (complete)
M00572 Pimeloyl-ACP biosynthesis, BioC-BioH pathway, malonyl-ACP => pimeloyl-ACP [PATH:map00780] (5) (1 block missing)
M00123 Biotin biosynthesis, pimeloyl-ACP/CoA => biotin [PATH:map00780] (4) (complete)
M00573 Biotin biosynthesis, BioI pathway, long-chain-acyl-ACP => pimeloyl-ACP => biotin [PATH:map00780] (4) (1 block missing)
M00577 Biotin biosynthesis, BioW pathway, pimelate => pimeloyl-CoA => biotin [PATH:map00780] (4) (1 block missing)
M00126 Tetrahydrofolate biosynthesis, GTP => THF [PATH:map00790 map00670] (5) (2 blocks missing)
M00121 Heme biosynthesis, glutamate => protoheme/siroheme [PATH:map00860] (12) (2 blocks missing)
M00118 Glutathione biosynthesis, glutamate => glutathione [PATH:map00480] (2) (complete)
M00140 C1-unit interconversion, prokaryotes [PATH:map00670] (2) (1 block missing)
M00141 C1-unit interconversion, eukaryotes [PATH:map00670] (1) (1 block missing)
Polyamine biosynthesis
M00134 Polyamine biosynthesis, arginine => ornithine => putrescine [PATH:map00330] (2) (complete)

Structural complex

Energy metabolism

ATP synthesis
M00144 NADH:quinone oxidoreductase, prokaryotes [PATH:map00190] (15) (complete)
M00149 Succinate dehydrogenase, prokaryotes [PATH:map00190] (4) (complete)
M00151 Cytochrome bc1 complex respiratory unit [PATH:map00190] (3) (complete)
M00152 Cytochrome bc1 complex [PATH:map00190] (3) (1 block missing)

M00154 Cytochrome c oxidase [PATH:map00190] (2) (1 block missing)
M00155 Cytochrome c oxidase, prokaryotes [PATH:map00190] (3) (complete)
M00153 Cytochrome d ubiquinol oxidase [PATH:map00190] (2) (complete)
M00157 F-type ATPase, prokaryotes and chloroplasts [PATH:map00190 map00195] (8) (complete)

Genetic information processing

DNA polymerase

M00260 DNA polymerase III complex, bacteria [PATH:map03030 map03430 map03440] [BR:ko03032 ko03400] (7) (complete)

RNA polymerase

M00183 RNA polymerase, bacteria [PATH:map03020] [BR:ko03021 ko03400] (4) (complete)

RNA processing

M00394 RNA degradosome [PATH:map03018] (3) (1 block missing)

Ribosome

M00178 Ribosome, bacteria [PATH:map03010] [BR:ko03011] (53) (complete)
M00179 Ribosome, archaea [PATH:map03010] [BR:ko03011] (31) (1 block missing)

Environmental information processing

Mineral and organic ion transport system

M00188 NitT/TauT family transport system [BR:ko02000] (2) (1 block missing)
M00299 Spermidine/putrescine transport system [PATH:map02010] [BR:ko02000] (4) (complete)
M00442 Putative hydroxymethylpyrimidine transport system [PATH:map02010] [BR:ko02000] (3) (complete)

Saccharide, polyol, and lipid transport system

M00669 gamma-Hexachlorocyclohexane transport system [BR:ko02000] (7) (complete)
M00210 Phospholipid transport system [PATH:map02010] [BR:ko02000] (8) (complete)
M00670 Mce transport system [BR:ko02000] (6) (1 block missing)

Phosphate and amino acid transport system

M00238 D-Methionine transport system [PATH:map02010] [BR:ko02000] (3) (complete)

Metallic cation, iron-siderophore and vitamin B12 transport system

M00244 Putative zinc/manganese transport system [BR:ko02000] (3) (complete)

ABC-2 type and other transport systems

M00250 Lipopolysaccharide transport system [PATH:map02010] [BR:ko02000] (2) (complete)
M00320 Lipopolysaccharide export system [PATH:map02010] [BR:ko02000] (3) (complete)
M00255 Lipoprotein-releasing system [PATH:map02010] [BR:ko02000] (2) (complete)
M00259 Heme transport system [PATH:map02010] [BR:ko02000] (3) (complete)
M00254 ABC-2 type transport system [BR:ko02000] (2) (complete)

Drug efflux transporter/pump

M00720 Multidrug resistance, efflux pump VexEF-TolC (1) (1 block missing)

Bacterial secretion system

M00325 alpha-Hemolysin/cyclolysin transport system [PATH:map02010 map03070] [BR:ko02044] (1) (1 block missing)
M00326 RTX toxin transport system [BR:ko02044] (1) (1 block missing)
M00571 AlgE-type Mannuronan C-5-Epimerase transport system [BR:ko02044] (1) (1 block missing)
M00339 RaxAB-RaxC type I secretion system [PATH:map04626] [BR:ko02044] (1) (1 block missing)
M00331 Type II general secretion system [PATH:map03070] [BR:ko02044] (1) (1 block missing)
M00333 Type IV secretion system [PATH:map03070] [BR:ko02044] (1) (1 block missing)
M00335 Sec (secretion) system [PATH:map03070] [BR:ko02044] (11) (complete)
M00429 Competence-related DNA transformation transporter [BR:ko02044] (2) (1 block missing)

Functional set

Metabolism

Aminoacyl tRNA

M00360 Aminoacyl-tRNA biosynthesis, prokaryotes [PATH:map00970] (20) (complete)
M00359 Aminoacyl-tRNA biosynthesis, eukaryotes [PATH:map00970] (20) (2 blocks missing)

Nucleotide sugar

M00362 Nucleotide sugar biosynthesis, prokaryotes [PATH:map00520] (5) (2 blocks missing)
M00361 Nucleotide sugar biosynthesis, eukaryotes [PATH:map00520] (4) (2 blocks missing)

Environmental information processing

Two-component regulatory system

M00453 QseC-QseB (quorum sensing) two-component regulatory system [PATH:map02020] [BR:ko02022] (3) (complete)

M00475 BarA-UvrY (central carbon metabolism) two-component regulatory system [PATH:map02020] [BR:ko02022] (1) (1 block missing)

Drug efflux transporter/pump

M00647 Multidrug resistance, efflux pump AcrAB-TolC/SmeDEF (5) (1 block missing)

M00699 Multidrug resistance, efflux pump AmeABC (4) (2 blocks missing)

Drug resistance

M00628 beta-Lactam resistance, AmpC system [PATH:map01501] (2) (2 blocks missing)

M00742 Aminoglycoside resistance, protease FtsH (4) (complete)

M00743 Aminoglycoside resistance, protease HtpX (1) (2 blocks missing)

M00729 Fluoroquinolone resistance, gyrase-protecting protein Qnr (1) (2 blocks missing)

M00721 Cationic antimicrobial peptide (CAMP) resistance, arnBCADTEF operon [PATH:map01503] (1) (2 blocks missing)

M00728 Cationic antimicrobial peptide (CAMP) resistance, envelope protein folding and degrading factors DegP and DsbA [PATH:map01503] (1) (2 blocks missing)

Signature module

Gene set

Drug resistance

M00651 Vancomycin resistance, D-Ala-D-Lac type [PATH:map01502 map02020] (1) (2 blocks missing)

M00718 Multidrug resistance, efflux pump MexAB-OprM (4) (2 blocks missing)

M00696 Multidrug resistance, efflux pump AcrEF-TolC (1) (2 blocks missing)

M00697 Multidrug resistance, efflux pump MdtEF-TolC (1) (2 blocks missing)
