# Supplementary material for: Candidate pathogenicity islands in the genome of ‘Candidatus Rickettsiella isopodorum’, an intracellular bacterium infecting terrestrial isopod crustaceans
Source: PeerJ. 2016 Dec 21;4:e2806. doi: 10.7717/peerj.2806 (PMC5181103; doi:10.7717/peerj.2806)
Supplement: Supplemental Information 2 [file peerj-04-2806-s002.zip › Supplement/blastkoala_results/BlastKOALA_Result_Summary.pdf]

BlastKOALA

Result

Help

Your BlastKOALA job

Query dataset: 1284 entries
Taxonomy group: Prokaryotes,Bacteria,Gammaproteobacteria (Taxonomy ID: 59195)
KEGG database searched: family_eukaryotes.pep,genus_prokaryotes.pep
Job submitted: Wed Oct 26 04:37:53 JST 2016
Job completed: Wed Oct 26 05:27:10 JST 2016

Annotation data View | Download

Summary 865 entries (67.4%) annotated

Functional category

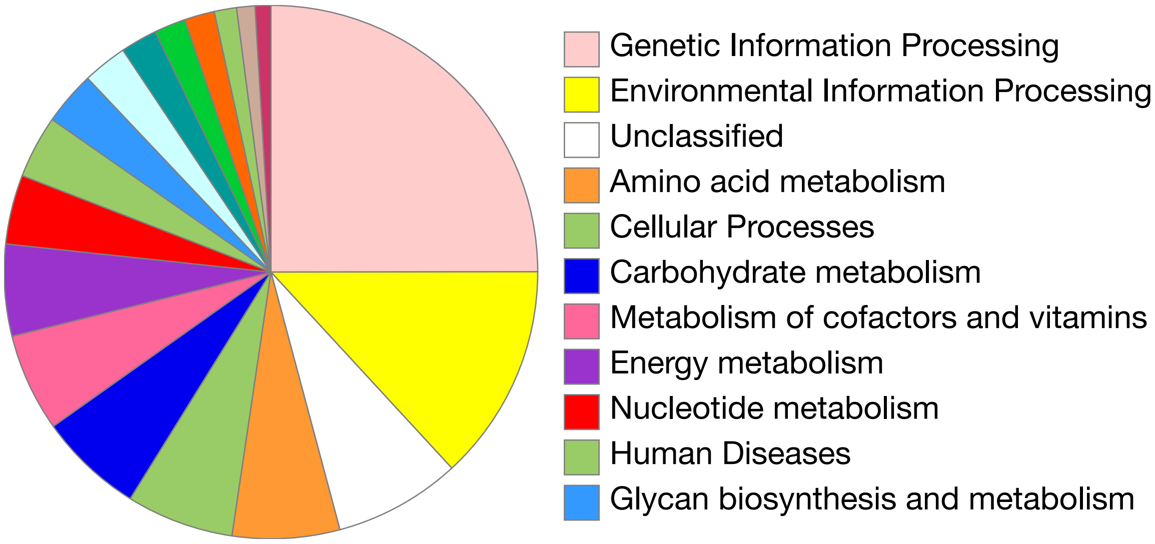

See color codes

KEGG Mapper Reconstruct Pathway
Reconstruct Brite
Reconstruct Module

[BlastKOALA | KEGG | Kanehisa Labs]
