# Supplementary figures and images for: Candidate pathogenicity islands in the genome of ‘Candidatus Rickettsiella isopodorum’, an intracellular bacterium infecting terrestrial isopod crustaceans
Source: PeerJ. 2016 Dec 21;4:e2806. doi: 10.7717/peerj.2806 (PMC5181103; doi:10.7717/peerj.2806)

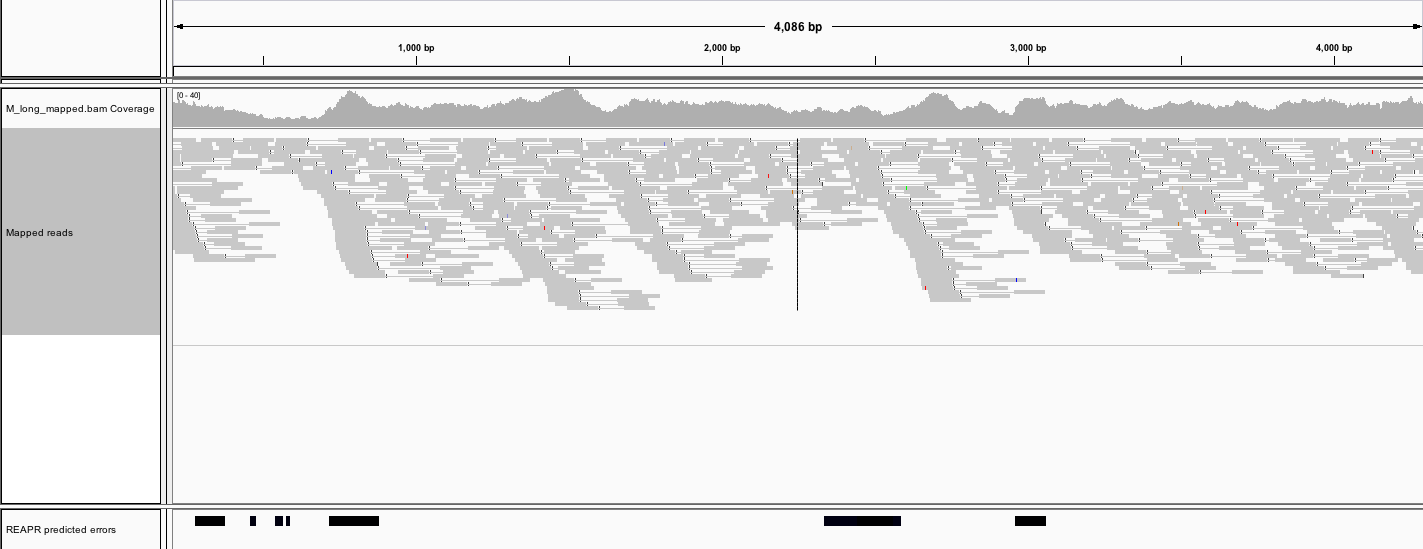

Supplement: Supplemental Information 2 [file peerj-04-2806-s002.zip › Supplement/assembly_eval/reapr_grylli_rathkei/contig_828.tiff]

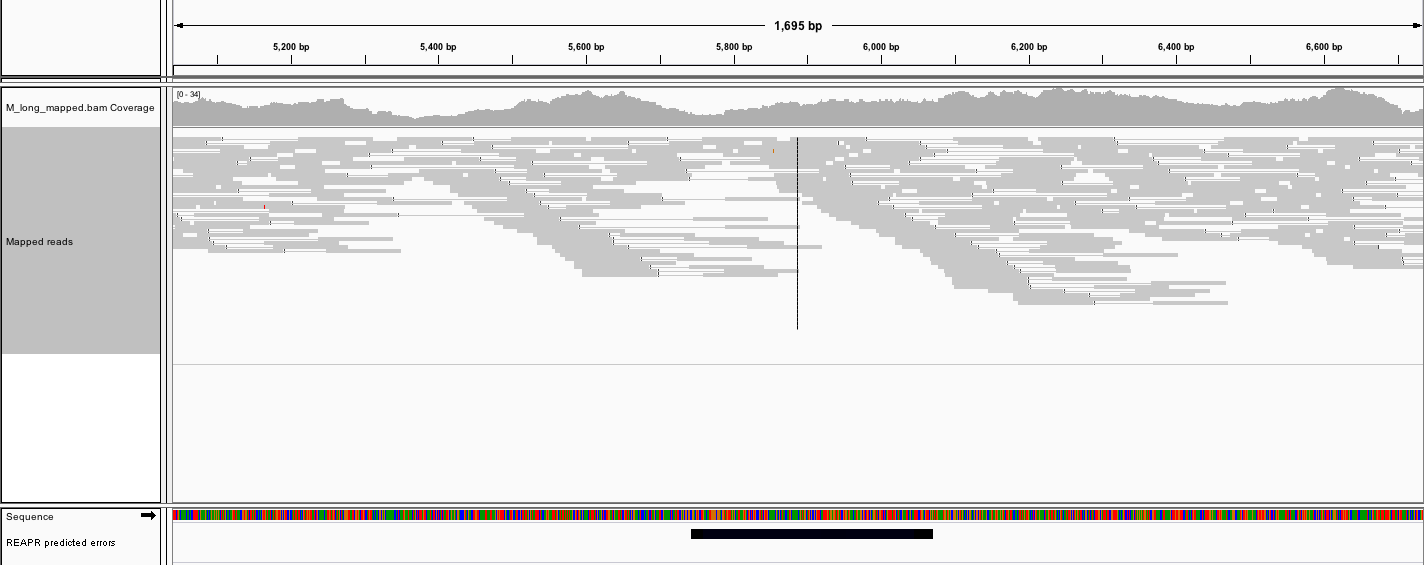

Supplement: Supplemental Information 2 [file peerj-04-2806-s002.zip › Supplement/assembly_eval/reapr_grylli_rathkei/contig_844.tiff]

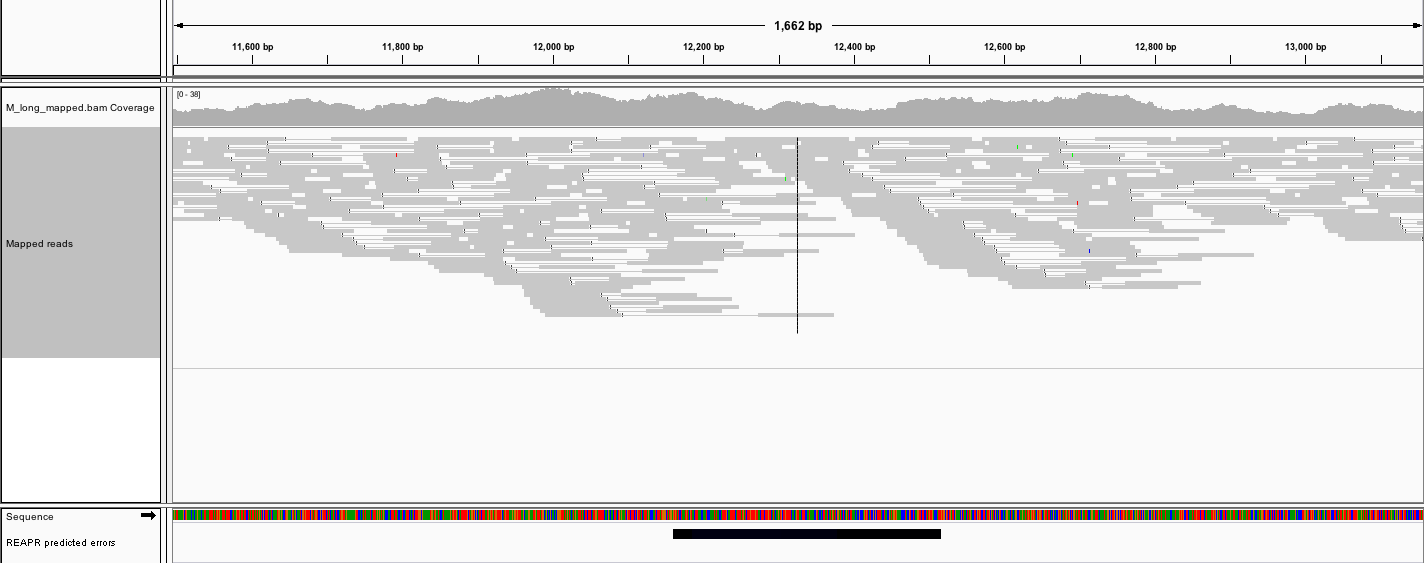

Supplement: Supplemental Information 2 [file peerj-04-2806-s002.zip › Supplement/assembly_eval/reapr_grylli_rathkei/contig_847.tiff]

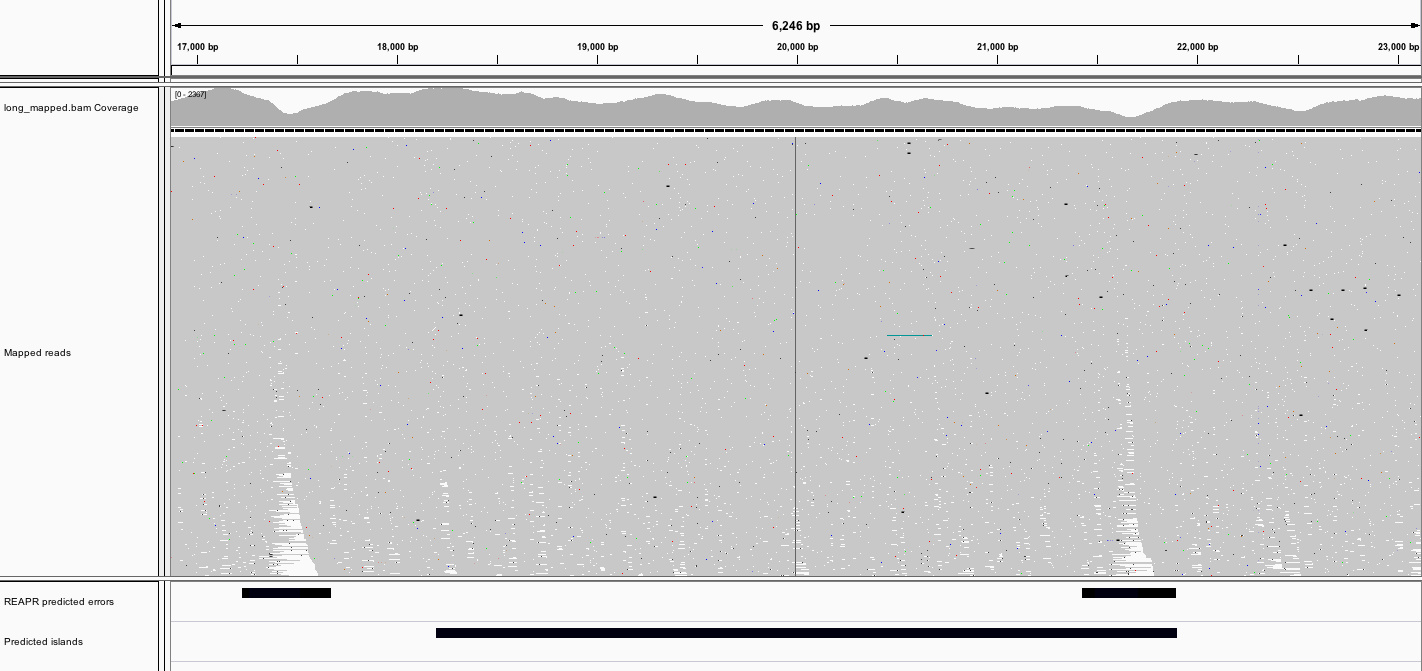

Supplement: Supplemental Information 2 [file peerj-04-2806-s002.zip › Supplement/assembly_eval/reapr_isopodorum/contig_191.tiff]

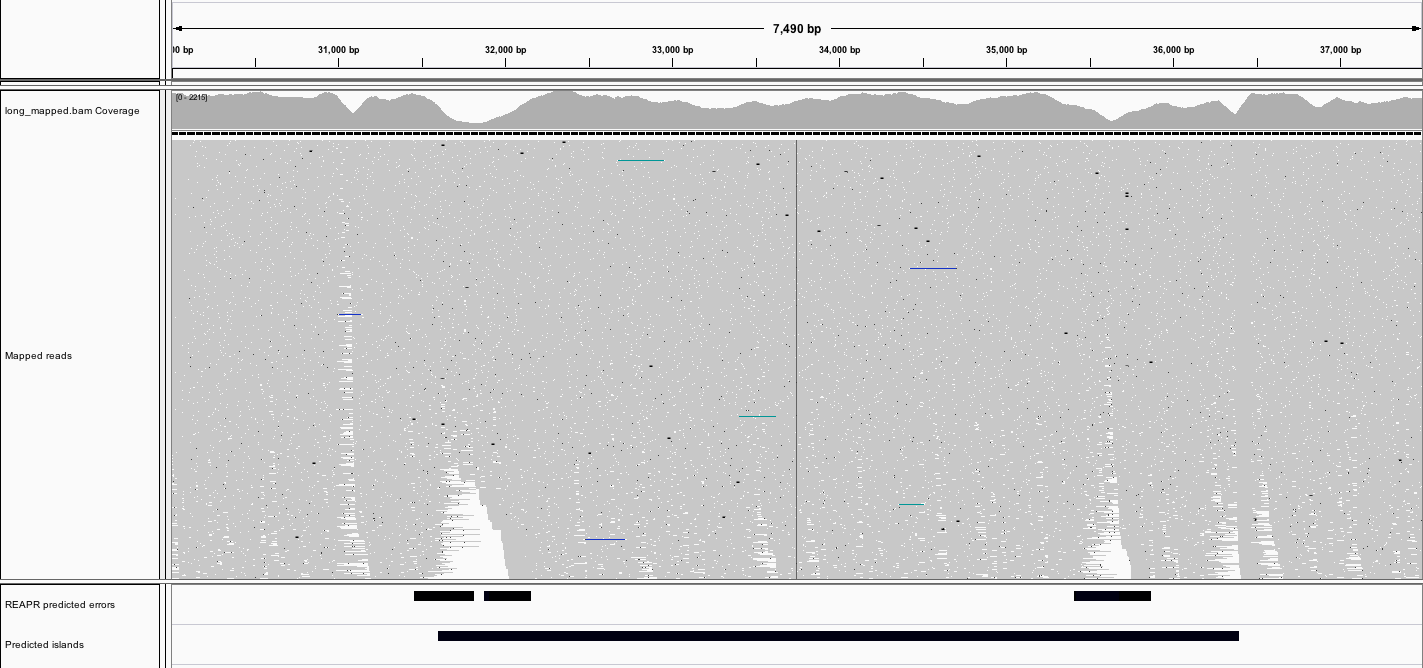

Supplement: Supplemental Information 2 [file peerj-04-2806-s002.zip › Supplement/assembly_eval/reapr_isopodorum/contig_193.tiff]

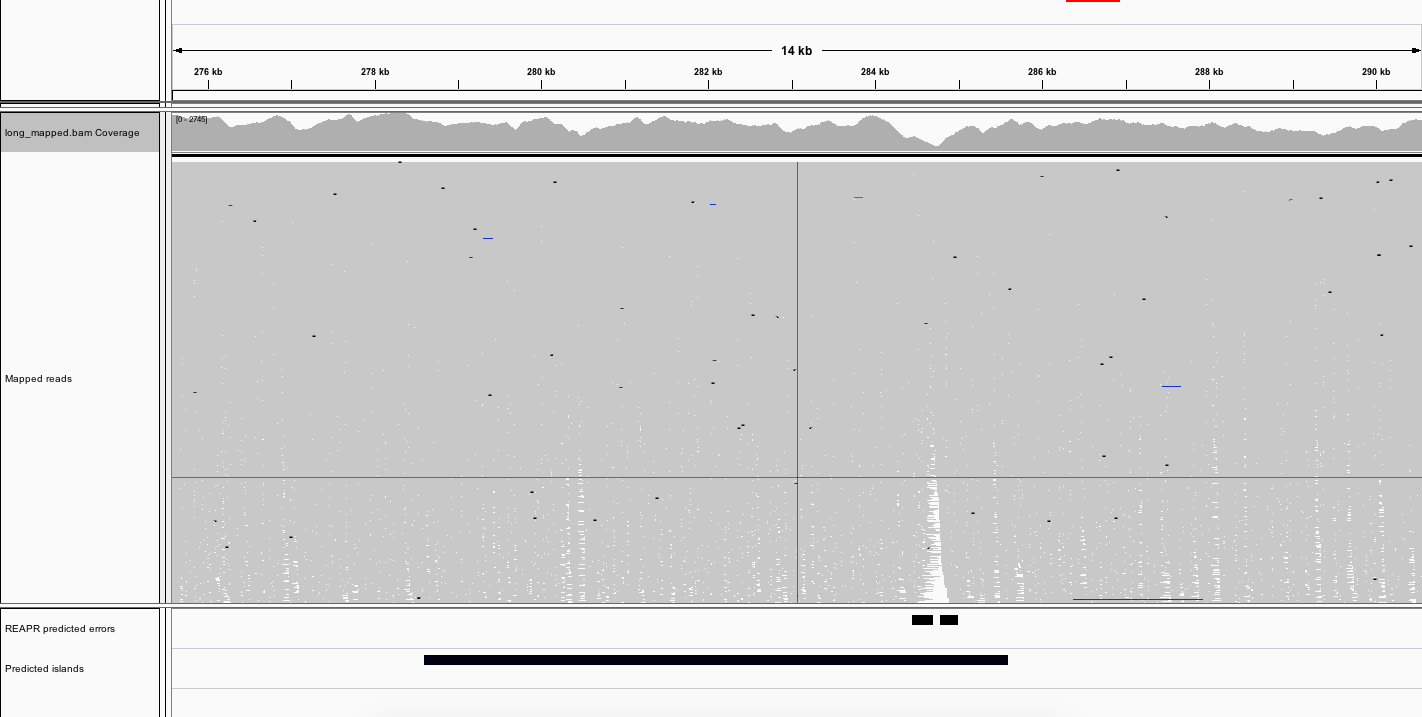

Supplement: Supplemental Information 2 [file peerj-04-2806-s002.zip › Supplement/assembly_eval/reapr_isopodorum/contig_197.tiff]

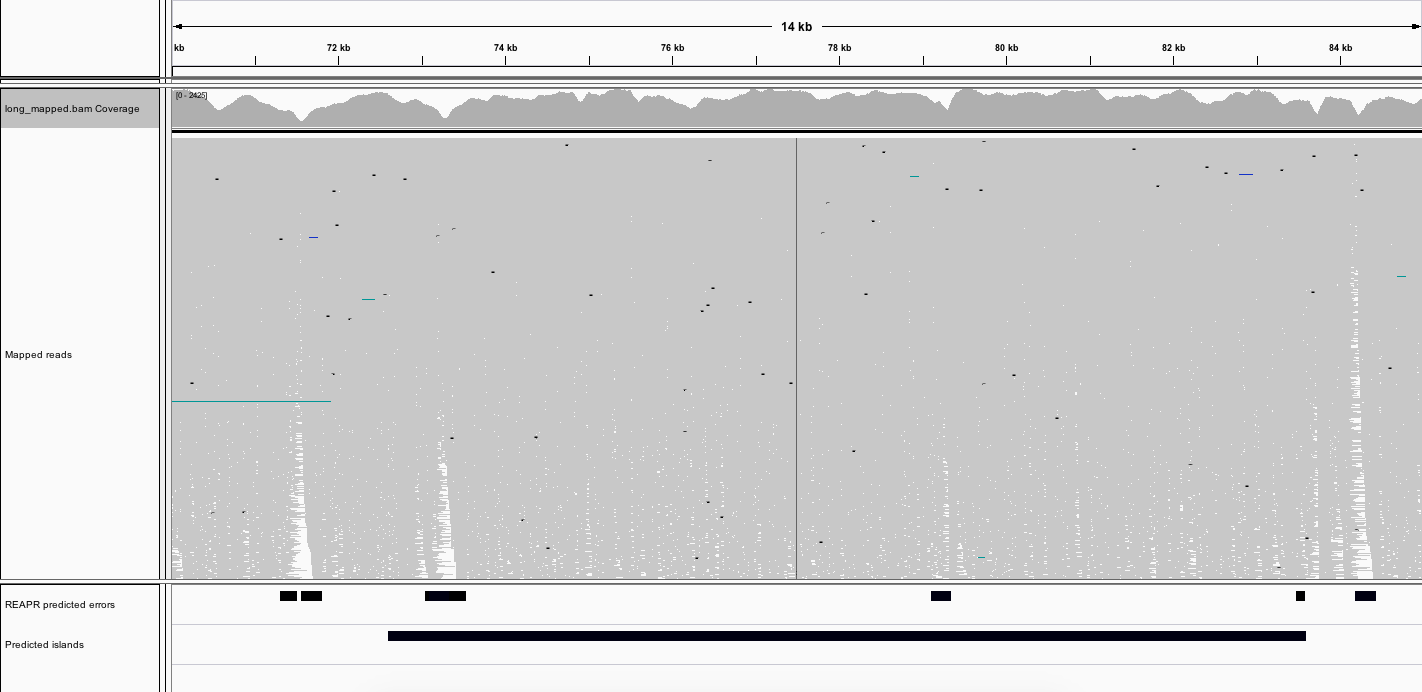

Supplement: Supplemental Information 2 [file peerj-04-2806-s002.zip › Supplement/assembly_eval/reapr_isopodorum/contig_197b.tiff]

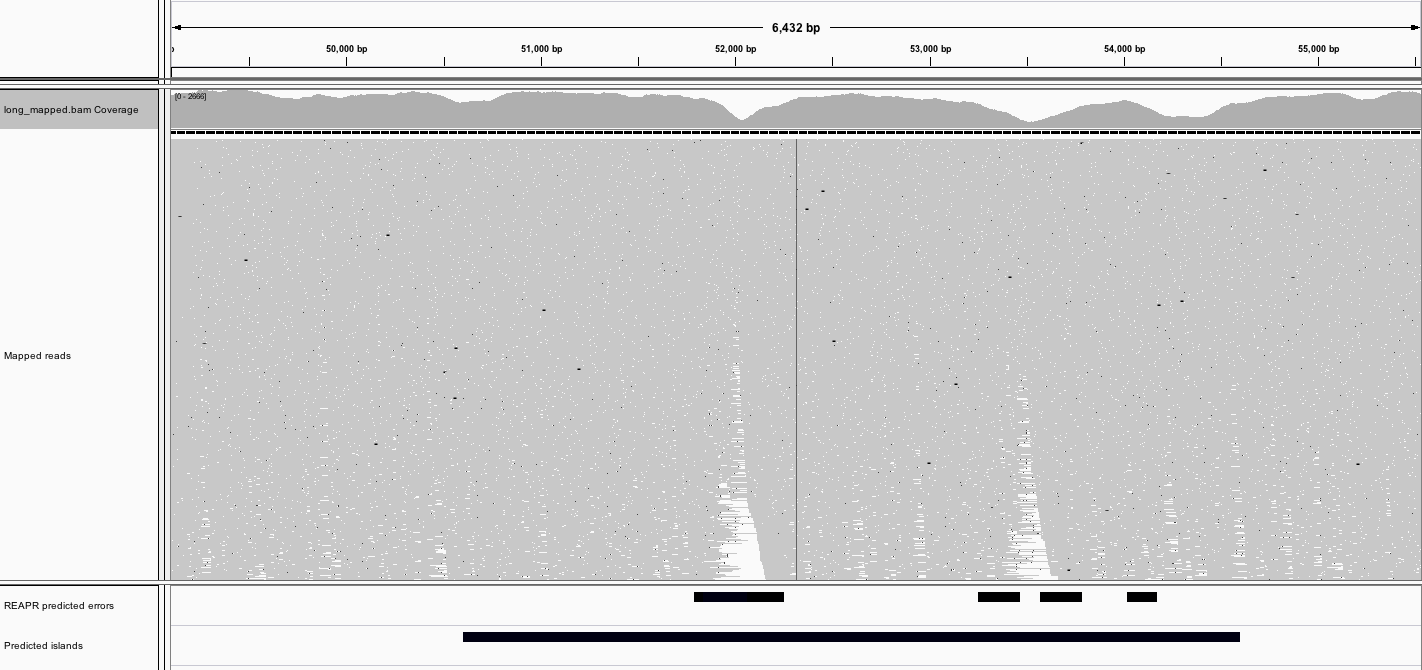

Supplement: Supplemental Information 2 [file peerj-04-2806-s002.zip › Supplement/assembly_eval/reapr_isopodorum/contig_197c.tiff]

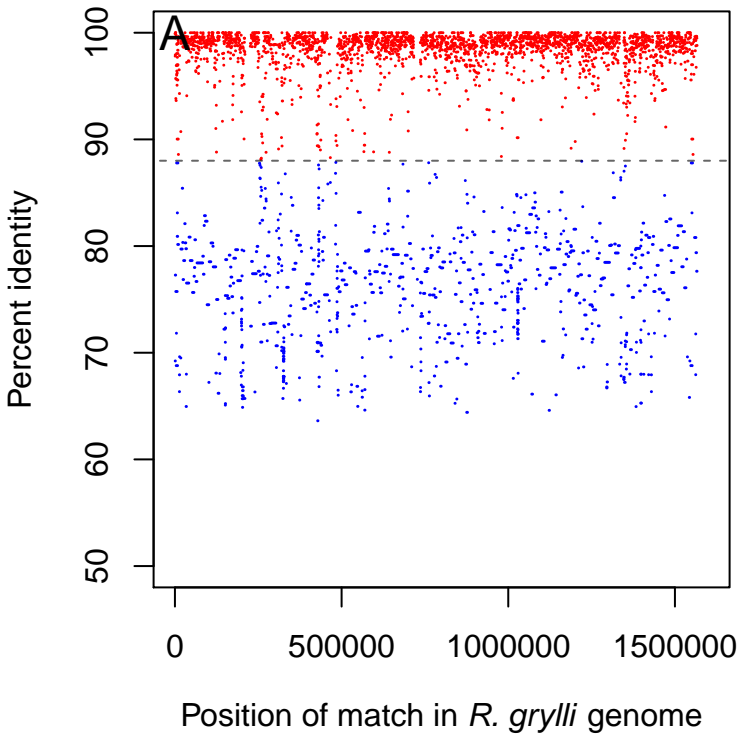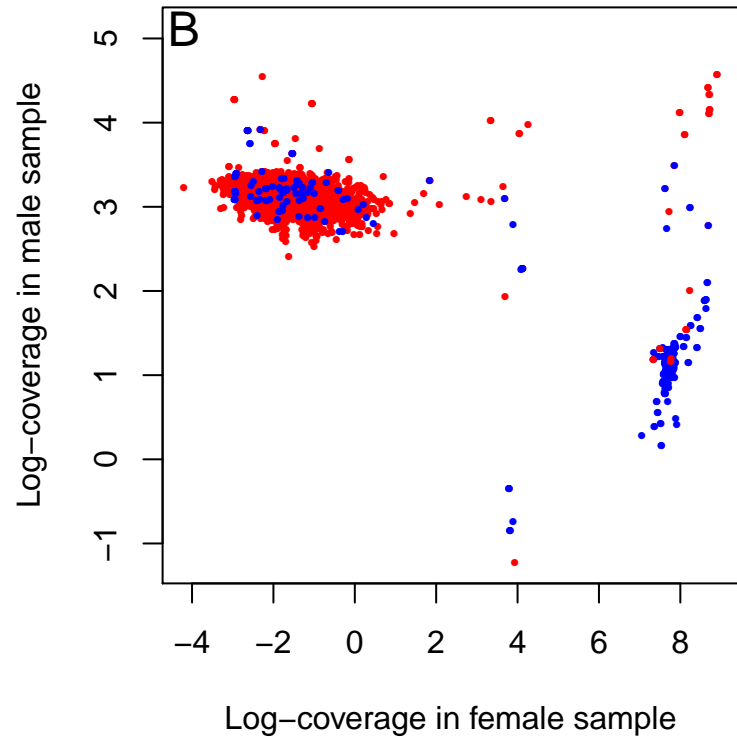

Supplement: Supplemental Information 2 [file peerj-04-2806-s002.zip › Supplement/assembly_scripts/Rickettsiella_matches.pdf]

*Diplorickettsia massiliensis*

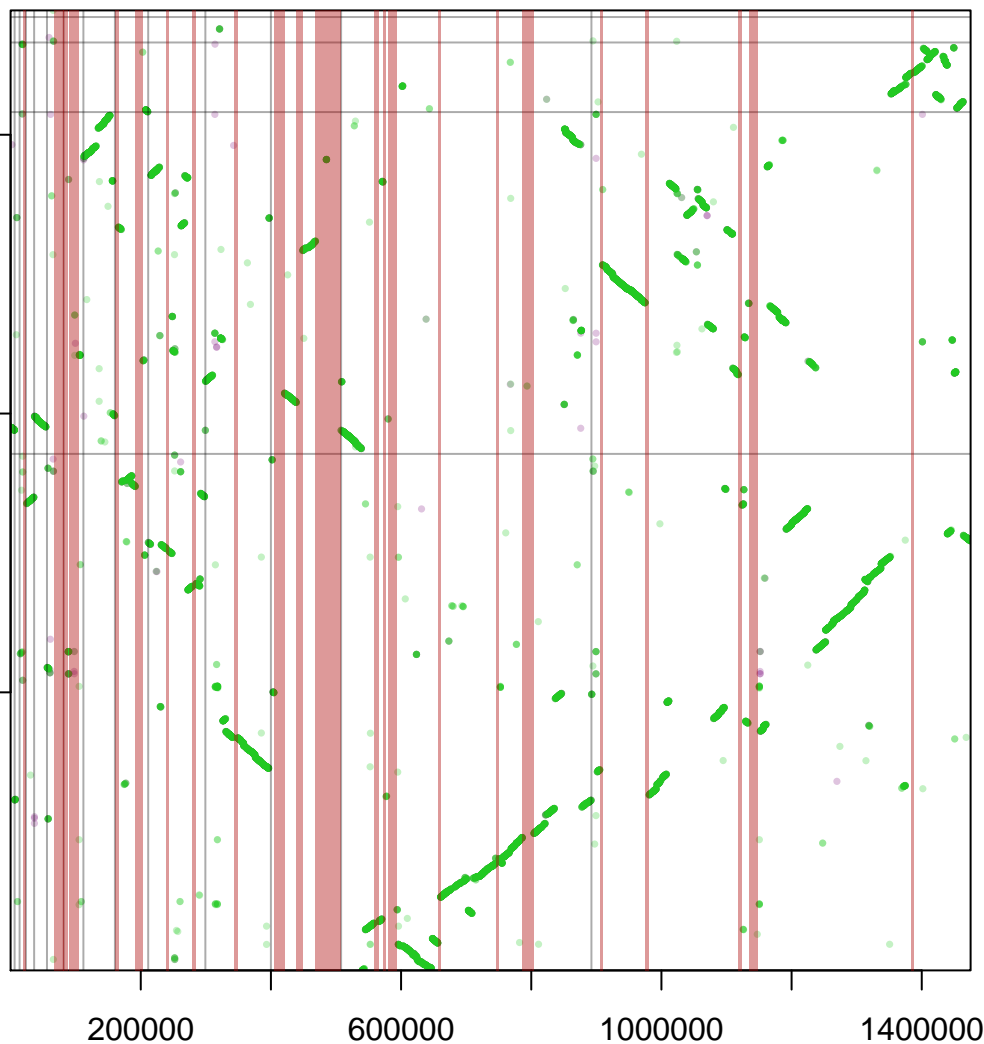

*Rickettsiella isopodorum*

Supplement: Supplemental Information 2 [file peerj-04-2806-s002.zip › Supplement/comparative_genomics/dot_plot_isopodorum_diplorickettsia_200/dot_plot_200.pdf]

Rickettsiella grylli

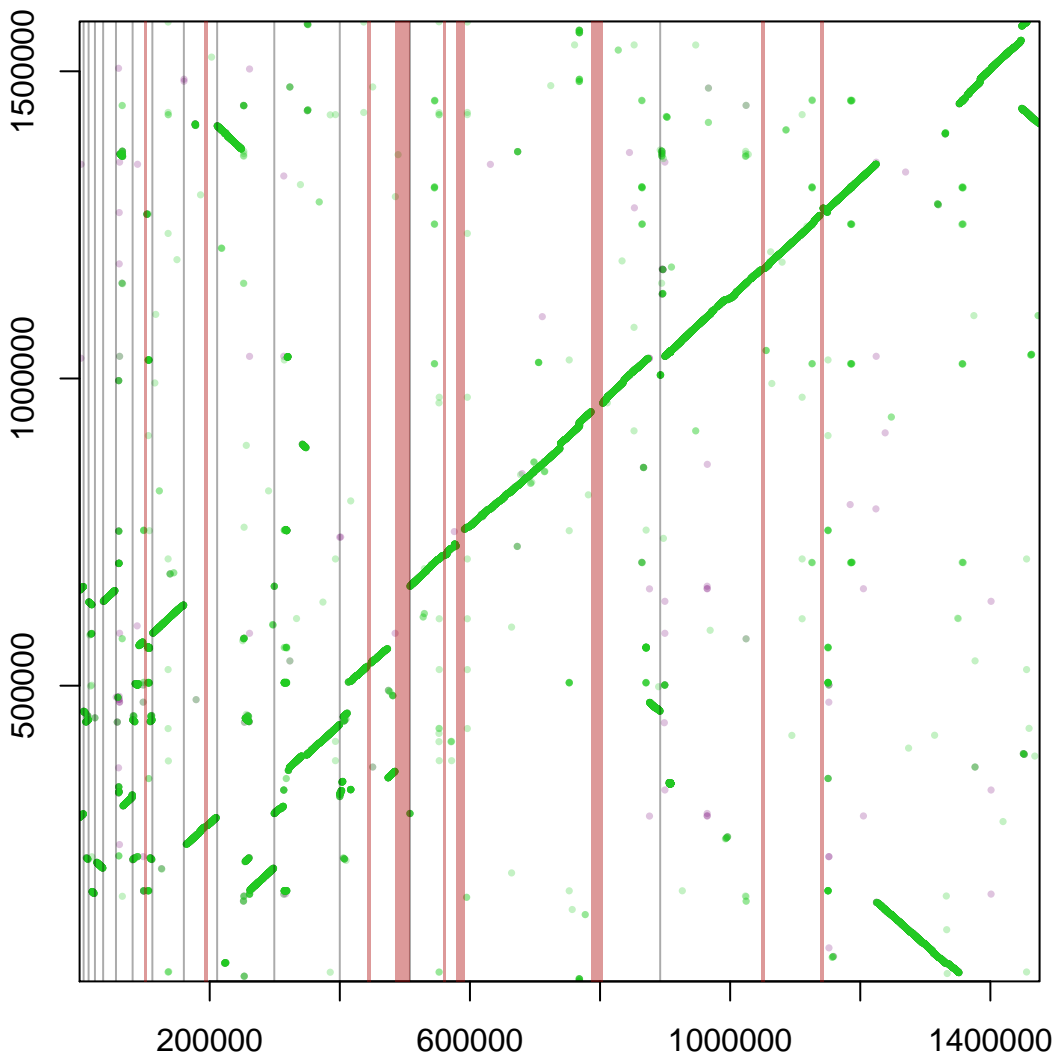

Rickettsiella isopodorum

Supplement: Supplemental Information 2 [file peerj-04-2806-s002.zip › Supplement/comparative_genomics/dot_plot_isopodorum_grylli_200/dot_plot_200.pdf]
